# Diversification in Functions and Expressions of Soybean *FLOWERING LOCUS T* Genes Fine-Tunes Seasonal Flowering

**DOI:** 10.3389/fpls.2021.613675

**Published:** 2021-04-26

**Authors:** Su Hyeon Lee, Cheol Woo Choi, Kyoung Mi Park, Wook-Hun Jung, Hyun Jin Chun, Dongwon Baek, Hyun Min Cho, Byung Jun Jin, Mi Suk Park, Dong Hyeon No, Lack Hyeon Lim, Sang In Shim, Jong Il Chung, Min Chul Kim

**Affiliations:** ^1^Division of Applied Life Science (BK21 Four), Plant Molecular Biology and Biotechnology Research Center, Gyeongsang National University, Jinju, South Korea; ^2^Institute of Agriculture and Life Science, Gyeongsang National University, Jinju, South Korea; ^3^Department of Agronomy, Gyeongsang National University, Jinju, South Korea

**Keywords:** soybean, flowering time, FLOWERING LOCUS T, photoperiods, functional diversification, soybean PEBP family

## Abstract

The proper timing of flowering in response to environmental changes is critical for ensuring crop yields. *FLOWERING LOCUS T* (*FT*) homologs of the phosphatidylethanolamine-binding protein family play important roles as floral integrators in many crops. In soybean, we identified 17 genes of this family, and characterized biological functions in flowering for ten *FT* homologs. Overexpression of *GmFT* homologs in *Arabidopsis* revealed that a set of *GmFT* homologs, including *GmFT2a/2b*, *GmFT3a/3b*, and *GmFT5a/5b*, promoted flowering similar to *FT*; in contrast, *GmFT1a/1b*, *GmFT4*, and *GmFT6* delayed flowering. Consistently, expressions of *GmFT2a*, *GmFT2b*, and *GmFT5a* were induced in soybean leaves in response to floral inductive short days, whereas expressions of *GmFT1a* and *GmFT4* were induced in response to long days. Exon swapping analysis between floral activator *GmFT2a* and floral repressor *GmFT4* revealed that the segment B region in the fourth exon is critical for their antagonistic functions. Finally, expression analysis of *GmFT2a*, *GmFT5a*, and *GmFT4* in soybean accessions exhibiting various flowering times indicated that the mRNA levels of *GmFT2a* and *GmFT5a* were higher in early flowering accessions than in late-flowering accessions, while *GmFT4* showed the opposite pattern. Moreover, the relative mRNA levels between *GmFT2a*/*GmFT5a* and *GmFT4* was important in determining day length-dependent flowering in soybean accessions. Taken together, our results suggest that the functions of *GmFT* homologs have diversified into floral activators and floral repressors during soybean evolution, and the timing of flowering in response to changing day length is determined by modulating the activities of antagonistic *GmFT* homologs.

## Introduction

Plants can sense seasonal changes, such as photoperiod and ambient temperature, and modulate their growth and development accordingly. This is especially important in crops, where the decision of the proper time for transition from vegetative to reproductive phases in response to changing environments is crucial to their adaptability to agricultural habitats and productivity. Soybean [*Glycine max* (L.) Merr.], a facultative short-day (SD) plant, is grown in a wide range of latitudes from the equator to 50° and cultivated in broad regions, including Asia, America, and Europe. Different soybean cultivars exhibit different flowering times and maturity according to their habitats (Watanabe et al., 2012). The wide adaptability of soybean plants to diverse environments has been acquired through genetic variations in a number of major genes that control flowering. To date, 11 major genes, *E1* through *E10* and *J*, have been identified as being involved in the control of flowering and maturity in soybean (Watanabe et al., 2012; Kong et al., 2014; Samanfar et al., 2017). Among these genes, *E6*, *E9*, and *J* promote flowering and maturity, whereas the other genes delay flowering.

In plants, phosphatidylethanolamine-binding proteins (PEBPs), such as FLOWERING LOCUS T (FT) and TERMINAL FLOWER 1 (TFL1), play important roles in modulation of flowering in addition to various developmental processes (Wickland and Hanzawa, 2015). In soybean, the roles of PEBP homologs have been identified in control of flowering and stem growth. Two *TFL1* homologs, *GmTFL1a*, and *GmTFL1b*, were the first isolated *PEBP* genes in soybean; *GmTFL1b* was identified as a candidate gene for the *Dt1* locus, which controls stem termination in soybean (Liu et al., 2010; Tian et al., 2010). In addition, at least 10 *FT* homologs have been identified in the soybean genome (Kong et al., 2010; Liu et al., 2018). Of the 10 *GmFT* homologs, *GmFT2a*, and *GmFT5a* are known to function as floral activators, which promote flowering under floral inductive SD conditions in soybean. These transcripts are more abundant in SD- than long-day (LD)-grown soybean leaves, and their ectopic expression in *Arabidopsis* and soybean promotes flowering (Kong et al., 2010; Sun et al., 2011). Recently, the soybean maturity gene *E9* was identified as *GmFT2a*. Delayed flowering as a result of the *e9* allele is due to the insertion of a *Ty-1*/*copia*-like retrotransposon in the first intron of *GmFT2a*, resulting in transcriptional repression (Zhao et al., 2016). In contrast, other *GmFT* homologs, *GmFT1a, and GmFT4*, function as floral repressors (Zhai et al., 2014; Liu et al., 2018). The expressions of *GmFT1a* and *GmFT4* are highly induced by LD, but suppressed by SD conditions. Their activation in LD conditions are dependent on functional *E1*, the key soybean maturity gene (Xia et al., 2012). Moreover, their expression is high in late-flowering soybean accessions. Overexpression of both *GmFT1a* and *GmFT4* delays flowering in transgenic *Arabidopsis* and soybean plants. These results suggest that both *GmFT1a* and *GmFT4* play critical roles in the suppression of soybean flowering under non-inductive LD conditions. Recently, *GmFT4* was identified as a possible candidate for the maturity locus *E10* (Samanfar et al., 2017). Taken together, these data suggest that the functions of *GmFT* genes have become diversified in controlling flowering time and maturity of soybean. Moreover, the relative transcript abundance of two antagonistic *GmFT* genes, *GmFT2a*/*5a* and *GmFT1a*/*4*, is important for determining the proper flowering time under diverse growth conditions. However, the roles of other *GmFT* homologs, such as *GmFT3a*/*b* and *GmFT6*, in soybean flowering and maturity remain unclear.

In addition to soybean, functional diversification in *FT* homologs has also been reported in other plant species, such as the sunflower (Blackman et al., 2010), sugar beet (Pin et al., 2010), onion (Lee et al., 2013), tobacco (Harig et al., 2012), sugarcane (Coelho et al., 2014), and longan (Winterhagen et al., 2013) plants. Wild alleles of three sunflower (*Helianthus annuus*) *FT* paralogs, *HaFT1*, *HaFT2*, and *HaFT4*, function as floral activators. However, a dominant-negative allele of *HaFT1* (*HaFT1-D*) containing a frame-shift mutation was selected during early domestication and *HaFT1-D* delays flowering by interfering with normal *HaFT4* function (Blackman et al., 2010). Sugar beets (*Beta vulgaris*) have two *FT* homologs, *BvFT1* and *BvFT2*. These two genes not only have opposite functions in flowering, but also display different expression patterns. *BvFT2* promotes flowering akin to *Arabidopsis FT*, and its expression is high in flowering-promoting conditions. In contrast, *BvFT1* represses flowering with higher expression levels in flowering-inhibiting conditions, such as before vernalization in the biennial sugar beet (Pin et al., 2010). In the onion (*Allium cepa*), six *FT* homologs have been identified (Lee et al., 2013). Overexpression of *AcFT1* and *AcFT2* in *Arabidopsis* promote flowering, while *35S::AcFT4* transgenic *Arabidopsis* plants demonstrate late-flowering. Moreover, *AcFT1* and *AcFT4* are also involved in LD photoperiod-dependent bulb formation, with opposite functions. The transcript levels of *AcFT1* and *AcFT4* are high in the leaves of onion plants before and after bulb formation, respectively. Overexpression of *AcFT1* in transgenic onion plants promotes bulb formation, but bulb formation is significantly delayed in *35S::AcFT4* onion plants. In addition, transgenic approaches in *Arabidopsis* revealed that *FT* homologs identified in other crop plants, including tobacco (*NtFT1*, *NtFT2*, and *NtFT3*), sugarcane (*ScFT1*), and longan (*DlFT2*), can also function as floral repressors (Harig et al., 2012; Winterhagen et al., 2013; Coelho et al., 2014). Taken together, these results suggest that in various crops, the functions of *FT* homologs have been diversified during evolution, and their floral transitions in response to environmental changes are tightly controlled by coordinated expressions and functions of *FT* family genes.

In the present study, we identified 17 soybean PEBP family genes, including ten *GmFT*, four *GmTFL1*, two *Brother of FT AND TFL1* (*GmBFT*), and a *Mother of FT AND TFL1* (*GmMFT*). We characterized the biological functions of these *GmFT* homologs in soybean flowering. Overexpression phenotypes in *Arabidopsis* and day length-dependent expression patterns of *GmFT* homologs suggest that a subset of these homologs, including *GmFT2a/2b*, *GmFT3a/3b*, and *GmFT5a/5b*, promote flowering in response to floral inductive SD conditions, while *GmFT1a/1b*, *GmFT4*, and *GmFT6* delay flowering in these conditions. By using exon swapping and amino acid substitution analyses, we characterized the structure-function relationship between floral activator *GmFT2a* and floral repressor *GmFT4*. Expression patterns of *GmFT* homologs in soybean accessions with various flowering times indicated that the relative cellular levels of floral activators, such as GmFT2a, GmFT5a, and a floral repressor, GmFT4, are critical factors in determining the day length-dependent flowering in soybean. Taken together, our results suggest that soybean plants regulate the timing of flowering in response to environmental conditions by modulating the activities of antagonistic *GmFT* homologs.

## Materials and Methods

### Plant Materials and Growth Conditions

*Arabidopsis* Col-0 plants were used in all experiments. *Arabidopsis* plants were grown at 23°C under either LD (16 h light/8 h dark) or SD (8 h light/16 h dark) conditions. The thirty-five soybean (*Glycine max*) accessions listed in Figure 7 were obtained from the United States Department of Agriculture Soybean Germplasm Collection. The twenty-four Korean soybean landraces listed in Supplementary Table 4 were obtained from the Rural Development Administration (RDA)-Genebank Information Center of Korea. For cDNA cloning and tissue-specific expression analyses, soybean plants (cv. Williams 82) were grown in the greenhouse during the normal growing season. For the day length-dependent gene expression analysis, soybean plants (cv. Williams 82) were grown in a growth chamber for 20 days under LD (16 h light/8 h dark) or SD (8 h light/16 h dark) conditions. The 35 USDA germplasms and 24 Korean soybean landraces used in this study were cultivated in the field during the natural growing season and the flowering time of each soybean line was determined from at least 15 individual plants of three years field experiments (three biological replicates).

### Isolation and Sequence Analysis of Soybean PEBP Family Members

Transcripts covering the entire coding regions of the 17 soybean PEBP family members were amplified from cDNAs synthesized from RNAs of various tissues of the Williams 82 cultivar by RT-PCR using gene-specific primer sets (Supplementary Table 1). PCR products were cloned and sequenced. The predicted amino acid sequences were aligned using the BioEdit program version 7.2.5^1^. The phylogenetic tree was constructed using the Neighbor-Joining method in the Mega 4 software program (Tamura et al., 2007) based on the amino acid sequence of the *Arabidopsis* and soybean PEBP family members.

### Gene Expression Analyses

Tissue-specific expression patterns were analyzed by RT-PCR and verified by subsequent Southern blotting. Total RNAs were isolated from various tissues at vegetative 1 (V1), vegetative 4 (V4), and reproductive 2 (R2) stages, and in developing seeds of Williams 82 plants grown in a natural green house. For diurnal expression analysis, the first trifoliate leaves were harvested every 4 h for 24 h from Williams 82 plants grown in a growth chamber for 20 days under LD (16 h light/8 h dark) or SD (8 h light/16 h dark) conditions. For the expression analysis of *GmFT2a*, *GmFT5a*, and *GmFT4* in various soybean accessions grown under field conditions, the third trifoliate (V3) leaves were sampled in bulk from at least three individual plants for each accession 30 DAS (V4 stage, before flowering). For the time course analysis of *GmFT2a*, *GmFT5a*, and *GmFT4* expression, both early flowering soybean accession (Williams 82) and late accession (PI229358) were grown under field conditions. The fully expended trifoliate leaves from the top of main stem were harvested from three independent plants from 20 to 100 DAS at 10 day intervals.

Total RNAs were isolated using LiCl precipitation (Verwoerd et al., 1989), and cDNA synthesis was performed using the SuperScript II Reverse Transcriptase (Invitrogen) according to the manufacturer’s instructions. In tissue-specific expression analysis, PCR products were separated by electrophoresis on 1% agarose gel and visualized by Southern blotting using [α-^32^P] dATP-labeled cDNA probes. Quantitative RT-PCR was performed in three independent biological replicates with a Bio-Rad CFX384^TM^ Real-time system. The expression of *GmPBB2* mRNA was used as a control to normalize the expression data. Data were analyzed with Bio-Rad CFX manager software (2*^–Δ^^Δ^^*Ct*^* method). The primers used for RT-PCR and quantitative RT-PCR are listed in Supplementary Table 2.

Correlation analysis between expression levels of *GmFT2a*, *GmFT5a*, and *GmFT4* and flowering times of various soybean accessions was carried out using R software^2^.

### Generation of Exon Swapping and Amino Acid Substitution Mutant Constructs

To construct chimeric genes which contained swapped exons or segment B regions between *GmFT2a* and *GmFT4*, we designed primers containing both *GmFT2a* and *GmFT4* sequences, such that the one end of each oligonucleotide contained the 3′-end sequence (10 nucleotides) of the exon/segment B of *GmFT2a* or *GmFT4*, whereas the other part contained the 5′-starting sequence of an adjacent exon/segment B of *GmFT2a* or *GmFT4*, respectively. After amplification of the appropriate fragments of *GmFT2a* and *GmFT4* cDNAs in the first round of PCR, each fragment was purified from the agarose gel, mixed, and used as template to obtain the full-length chimeric gene. Substitutions of single amino acids were performed using the QuickChange Site-directed Mutagenesis Kit (Clontech) according to the manufacturer’s instructions. DNA sequences of chimeric genes and amino acid substitution mutants were verified by sequencing. The primers used for exon swapping and amino acid substitution are listed in Supplementary Tables 5, 6, respectively.

### Ectopic Expression of *GmFTs* in *Arabidopsis*

The overexpression vectors for *GmFT* genes were constructed by cloning the full-length coding sequence of wild-type and mutant (chimeras and substitution) *GmFT* genes downstream of the CaMV 35S promoter in the pBJ36 vector (Gleave, 1992), and then these cassettes were shuttled into pMLBART. *Arabidopsis* Col-0 plants were transformed by the floral dip method (Clough and Bent, 1998). Transformants were selected on the soil by spraying Basta twice. Expression of transgenes was confirmed by RT-PCR.

### Accession Numbers

The cDNA sequences for 17 soybean PEBP family members reported in this paper have been deposited in the GenBank database with accession numbers KJ607990 (*GmFT1a*), KJ607991 (*GmFT1b*), KJ607992 (*GmFT2a*), KJ607993 (*GmFT2b*), KJ607994 (*GmFT3a*), KJ607995 (*GmFT3b*), KJ607996 (*GmFT4*), KJ607997 (*GmFT5a*), KJ607998 (*GmFT5b*), KJ607999 (*GmFT6*), KJ608000 (*GmBFTa*), KJ608001 (*GmBFTb*), KJ608002 (*GmMFT*), KJ608003 (*GmTFL1a*), KJ608004 (*GmTFL1b*), KJ608005 (*GmTFL1.2a*), and KJ608006 (*GmTFL1.2b*).

## Results

### Identification of Soybean PEBP Family Members

**FIGURE 1:**
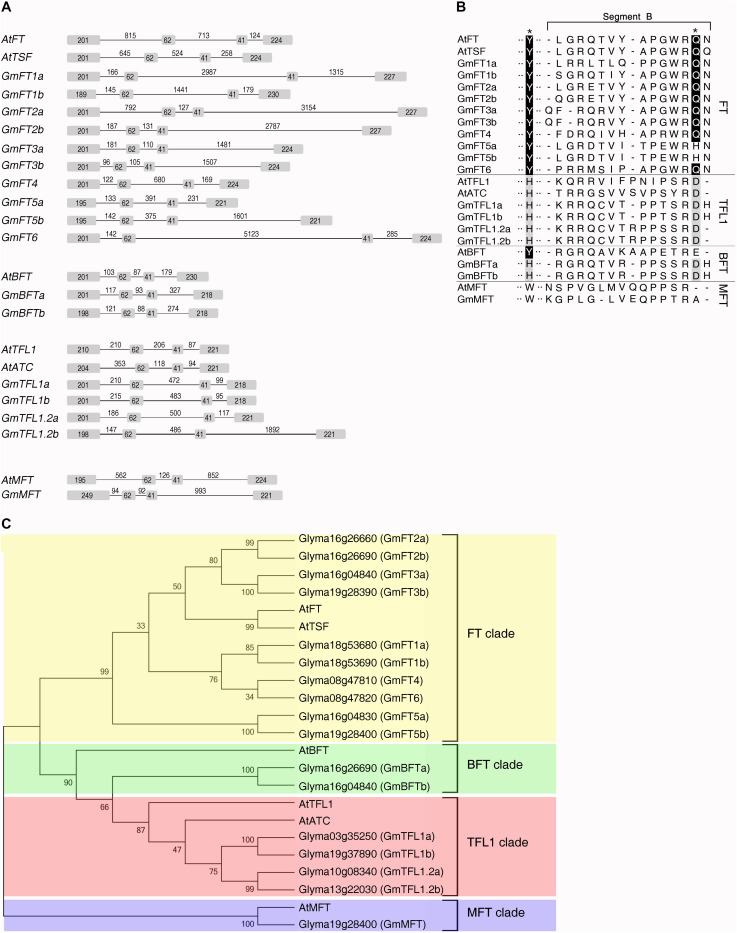
Identification and sequence analysis of soybean PEBP family members. **(A)** Genomic organization of the soybean and *Arabidopsis* PEBP family members. Boxes and lines represent exonic and intronic regions, respectively. Numbers indicate the length of exons and introns (base pairs). The gene structures of soybean PEBP family members were determined on the basis of the alignment between the genomic and cDNA sequences. **(B)** Phylogenetic analysis of *Arabidopsis* and soybean PEBP family members. The phylogenetic tree was constructed using the Neighbor-Joining method of Mega 4 software program (Tamura et al., 2007) based on the amino acid sequences of the *Arabidopsis* and soybean PEBP family members. Bootstrap values (1,000 replicates) are indicated at the branches of the tree. **(C)** Partial amino acid sequence alignment of the 14-amino acid segment B region of soybean and *Arabidopsis* PEBP family members. Black stars above the upper row indicate the Tyr85(Y)/His88(H) and Gln140(Q)/Asp144(D) residues specifying *Arabidopsis* FT and TFL1 functions in flowering, respectively.

To identify PEBP family members in soybean, we screened the Williams 82 genomic database^3^ with the amino acid sequence of *Arabidopsis* FT and identified 17 soybean gene models with sequence similarity to the entire coding region (Figure 1A). Based on the sequence of each gene model, we designed gene-specific primer pairs corresponding to each of the putative 17 soybean PEBP family members (Supplementary Table 1). RNA was extracted from soybean plants (cv Williams 82) grown in green house conditions, and these gene-specific primers were used to amplify the full-length cDNAs obtained by reverse-transcription (RT)-PCR. The nucleotide sequences of cloned cDNAs for these 17 soybean PEBP family members were determined by sequencing, and their corresponding amino acid sequences were deduced.

Phylogenetic analysis and alignment of amino acid sequences of *Arabidopsis* and soybean PEBP family members indicated that these 17 soybean orthologs fall into four different clades: the FT, BFT, TFL1, and MFT clades (Figure 1B and Supplementary Figure 1). Ten soybean genes belonging to the FT clade are further classified into 3 subclades. Among the ten soybean *FT* genes (*GmFTs*), *GmFT3a*/*b* and *GmFT2a*/*b* clustered together with *Arabidopsis FT* and *TSF* genes, which function as floral activators. The second subclade contains four *GmFT* genes, *GmFT1a*/*b*, *GmFT4*, and *GmFT6*. The remaining pair of *GmFT* genes, *GmFT5a* and *GmFT5b*, belongs to the third subclade. There are two pairs of *TFL1* homologs in soybean genome. One pair of TFL1 homologs was recently identified and named *GmTFL1a* and *GmTFL1b*, respectively, and fine-mapping analysis revealed *GmTFL1b* as a candidate gene for the soybean determinate stem (*Dt1*) locus (Liu et al., 2010; Tian et al., 2010). We named the second pair of *TFL1* homologs, *Glyma10g08340* and *Glyma13g22030*, *GmTFL1.2a* and *GmTFL1.2b*, respectively (Figure 1B). We also identified two *BFT* homologs and one *MFT* homolog in soybean genome, and named these *GmBFTa*, *GmBFTb*, and *GmMFT*, respectively (Figure 1B). Phylogenetic analysis indicated that only 3 genes of these 17 soybean orthologs, *GmMFT*, *GmFT4*, and *GmFT6*, are singletons, while the other 14 genes exist as pairs of homologs, reflecting the recent soybean whole-genome duplication event (Shoemaker et al., 2006).

The closely related FT and TFL1 proteins have opposite functions in the regulation of flowering: FT promotes flowering, while TFL1 represses flowering (Bradley et al., 1997; Ohshima et al., 1997; Ratcliffe et al., 1998; Kardailsky et al., 1999; Kobayashi et al., 1999). Initial analyses of the relationship between the structure and the function of closely related FT and TFL1 proteins identified two critical amino acid residues responsible for the opposite functions of *Arabidopsis* FT and TFL1, Tyr85/Gln140 in FT versus His88/Asp144 in TFL1 (Hanzawa et al., 2005; Ahn et al., 2006). These two amino acids are highly conserved in all soybean FT and TFL1 homologs except two, GmFT5a and GmFT5b, which have a His residue at the position corresponding to Gln140 of *Arabidopsis* FT (Figure 1C). The main difference between *Arabidopsis* FT and TFL1 is a 14-amino acid stretch forming an external loop in the crystal structures of these two proteins, called segment B of exon 4. This region is highly conserved in *FT* homologs, but selection in *TFL1* homologs has relaxed, leading to very divergent sequences (Ahn et al., 2006). Segment B has also been shown to be the critical difference in two beet FT homologs with opposite functions, BvFT1 and BvFT2 (Pin et al., 2010). GmFT2a shows the highest sequence similarity to *Arabidopsis* FT among the 10 soybean FT homologs, while GmFT1a/b, GmFT4, and GmFT6, belonging to a separate FT subclade, display higher sequence diversity (Figure 1C).

### Spatiotemporal Expression of Soybean PEBP Family Genes

**FIGURE 2:**
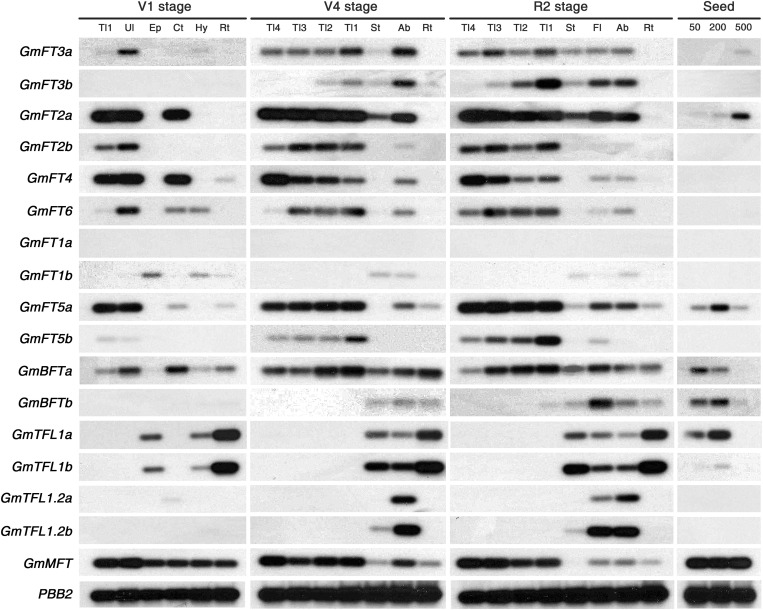
Expression analysis of soybean PEBP family members. Total RNAs were extracted from various tissues at different developmental stages of soybean plants grown in natural green house conditions. Transcript levels were analyzed by RT-PCR and subsequent Southern blotting. Soybean *PBB2* (20S proteasome beta subunit) mRNA (Glyma14g01850) was used as a control (Thakare et al., 2010). Tl: trifoliate leaf (Tl1; oldest, Tl4; youngest), Ul: unifoliate leaf, Ep: epicotyl, Ct: cotyledon, Hy: hypocotyl, Rt: root, St: stem, Ab: axillary bud, Fl: flower. Seed weights of 50, 200, and 500 mg are weights of single seeds.

Expression patterns of the 17 soybean PEBP family members were analyzed in various tissues and at different developmental stages of soybean plants grown in green house conditions. The transcript levels of 17 soybean PEBP genes were determined by RT-PCR using gene-specific primers (Supplementary Table 2). Since the transcripts of some PEBP genes, such as *GmFT1a* and *GmFT1b*, hardly detected on the gel, we performed subsequent Southern blot analysis to detect transcripts more easily and clearly (Figure 2). The transcripts of most of the *GmFT* genes accumulated abundantly in leaf tissues, such as the unifoliate leaf from the V1 stage and trifoliate leaves from both V4 and R2 stage plants, where light sensing primarily occurs. The transcripts of a pair of duplicated genes, *GmFT1a* and *GmFT1b*, were expressed at a very low level in most tissues examined, but *GmFT1b* was specifically expressed in stem tissues, including the epi- and hypocotyl at the V1 stage and the whole stem at later stages. In contrast to *GmFT* genes, *GmTFL1* genes were not expressed in the leaves; the transcripts of both *GmTFL1a* and *GmTFL1b* genes were highly expressed in roots and stems and moderately in flowers and axillary buds. Another homologous pair of *GmTFL1* genes, *GmTFL1.2a* and *GmTFL1.2b*, was specifically expressed in axillary buds and flowers. The expression of *GmTFL1* homologs in flowers was further confirmed by quantitative real-time (qRT)-PCR (Supplementary Figure 2) and this result was consistent with previous report showing the *GmTFL1* expression in flower (Tian et al., 2010). Moreover, each homologous pair of *GmTFL1* genes showed very similar spatiotemporal expression patterns, suggesting conservation of the regulation of gene expression of *GmTFL1* homologous pairs during the genome duplication. *GmBFTa* and *GmMFT* transcripts were detected in all tissues at most of the growth stages, but *GmBFTb* was expressed in relatively late stages of soybean plant growth. Interestingly, some of the soybean PEBP homologous genes, such as *GmFT2a*, *GmFT3a*, *GmFT5a*, *GmBFTs*, *GmTFL1s*, and *GmMFT*, were expressed in developing seeds, suggesting a possible role in seed development and maturation (Figure 2). Recently, it was reported that *Arabidopsis MFT* regulates seed germination through the ABA and GA signaling pathways (Xi et al., 2010). The overall expression patterns of soybean *FT* and *TFL1* homologs suggest that the biological functions of *GmFT* genes are likely more diverse than those of *GmTFL1* genes. Based on these results, we focused our efforts on determining the biological functions of *GmFT* homologs in soybean flowering.

### Ectopic Expression of *GmFT* Genes Differentially Affected Flowering Time in *Arabidopsis*

**FIGURE 3:**
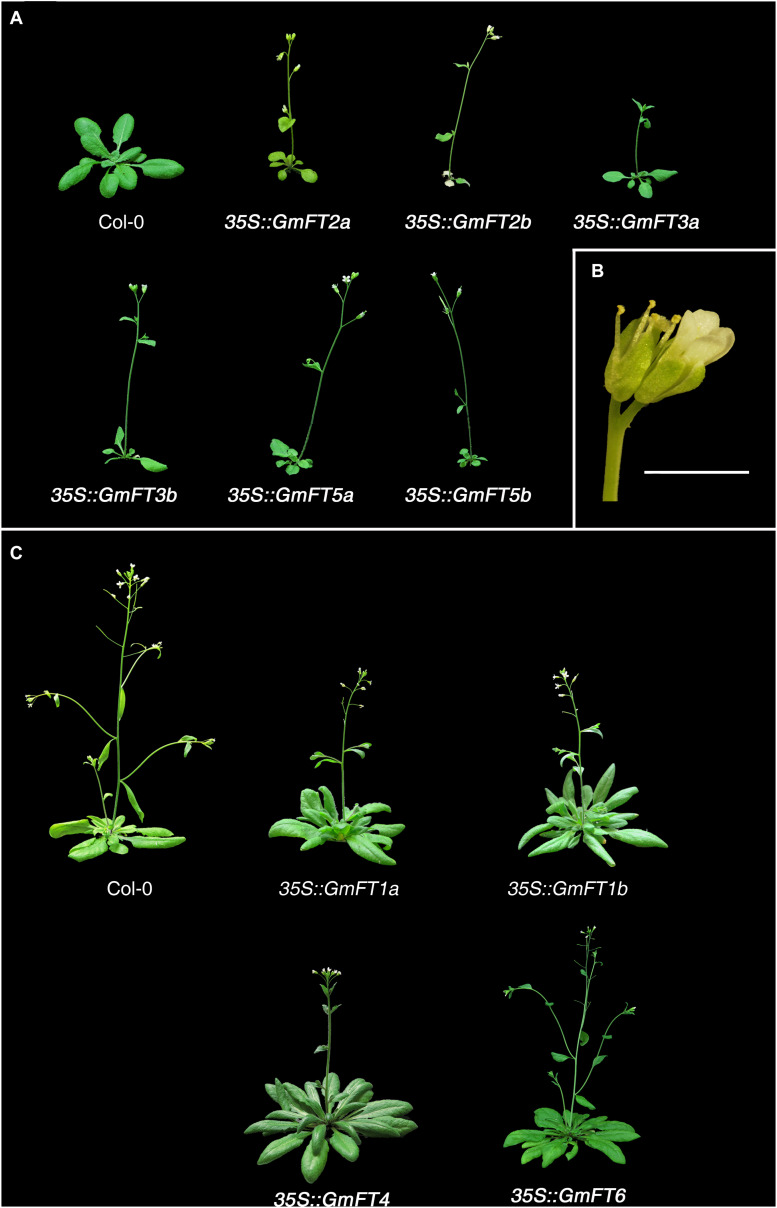
Effects of the ectopic expression of *GmFT* genes on flowering in transgenic *Arabidopsis* plants. **(A)** Phenotypes of 23-day old wild-type (Col-0) and transgenic *Arabidopsis* plants expressing soybean *GmFT2a, GmFT2b, GmFT3a, GmFT3b, GmFT5a*, and *GmFT5b*. **(B)** Phenotype of terminal flowers of *35S::GmFT2a*-expressing *Arabidopsis* plants. Scale bar is 2 mm. **(C)** Phenotypes of 40-day old wild-type and transgenic *Arabidopsis* plants expressing *GmFT1a*, *GmFT1b*, *GmFT4*, and *GmFT6*. Wild-type and T1 transgenic plants were grown on the soil at 23°C under long-day conditions.

In order to begin to determine the roles of *GmFT* genes in soybean flowering, we ectopically expressed soybean *FT* genes in *Arabidopsis* accession Columbia (Col-0) under the control of the constitutive cauliflower mosaic virus (CaMV) 35S promoter. The ectopic expression of *GmFT*s was confirmed by RT-PCR with gene-specific primers (data not shown; gene-specific primers used for this experiment are listed in Supplementary Table 2). Flowering time was determined in T1 plants. We used at least 3 independent T1 lines for each *GmFT* gene and more than 20 plants for the analysis of flowering time of *GmFTs* overexpressing plants (Table 1 and Supplementary Table 3). Overexpression of *GmFT2a*/*b*, *GmFT3a*/*b*, or *GmFT5a*/*b* in *Arabidopsis* strongly promoted flowering (Figure 3A, Table 1, and Supplementary Table 3). In addition, the growth of most of the primary inflorescence terminated in two or three terminal flowers, and secondary inflorescences were converted into solitary flowers (Figure 3B). However, overexpression of another subset of soybean *FT* homologs, including *GmFT1a*, *GmFT1b*, *GmFT4*, and *GmFT6*, repressed flowering of *Arabidopsis* plants under LD conditions, which otherwise promoted early flowering (Figure 3C and Table 1). Among them, *GmFT4* exhibited the strongest floral repressor activity. These results suggest that even though *GmFT* genes share structural and sequence similarity with *Arabidopsis FT*, their biological functions have differentially evolved following the genome duplication event.

**TABLE 1:** Flowering times determined by leaf number in long-day conditions.

Genotype	RLN^*a*^	CLN^*b*^	*n*
Wild-type, Col-0	10.7 ± 1.18	2.6 ± 0.50	20
*35S:GmFT2a*	3.3 ± 0.49	2.2 ± 0.42	23
*35S:GmFT2b*	2.9 ± 0.28	1.9 ± 0.33	37
*35S:GmFT3a*	5.7 ± 1.44	3.2 ± 0.86	28
*35S:GmFT3b*	3.0 ± 0.17	2.1 ± 0.78	33
*35S:GmFT5a*	2.9 ± 0.42	1.8 ± 0.72	35
*35S:GmFT5b*	3.1 ± 0.53	1.6 ± 0.56	35
*35S:GmFT1a*	18.2 ± 2.84	4.5 ± 1.12	35
*35S:GmFT1b*	14.5 ± 1.93	4.3 ± 1.12	35
*35S:GmFT4*	27.2 ± 6.08	7.0 ± 2.05	24
*35S:GmFT6*	19.4 ± 7.33	8.4 ± 5.22	32

*^a^Rosette leaf number. ^b^Cauline leaf number.*

### Differential Expression of *GmFT* Genes in Response to Day Length

**FIGURE 4:**
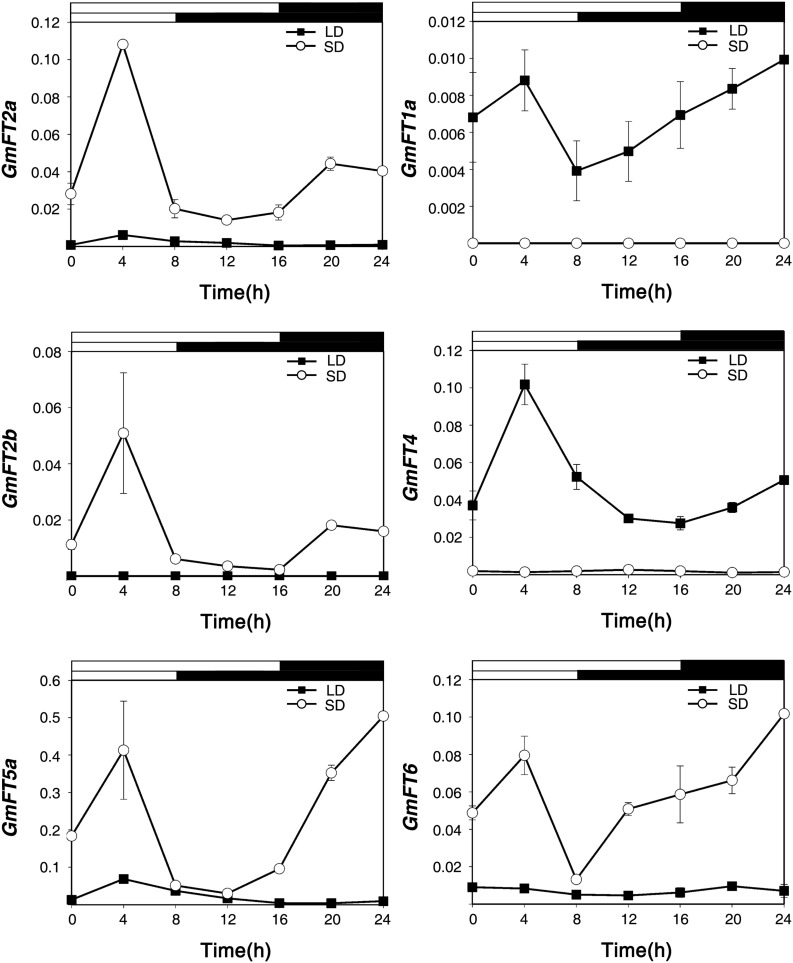
Diurnal expression of *GmFT* genes under LD and SD conditions. Total RNAs were extracted every 4 h from the first trifoliate leaves of 20-day old LD- and SD-grown plants, respectively. Relative mRNA levels of *GmFT* genes were analyzed by quantitative real-time PCR with three independent biological replicates and normalized to *GmPBB2* mRNA. White and dark bars indicate light and dark phases, respectively. Data are shown as means ± standard deviation.

It has been shown previously that the expression of *FT* is induced in response to floral inductive day length (Kardailsky et al., 1999; Kobayashi et al., 1999; Kojima et al., 2002; Valverde et al., 2004). To confirm the functional diversification of *GmFT* genes in soybean, we first analyzed their diurnal expression patterns in response to LD and SD conditions, and floral repressive and inductive day-length, respectively. Soybean plants (cv. Williams 82) were grown in a growth chamber for 20 days under LD (16 h light/8 h dark) or SD (8 h light/16 h dark) conditions, and the first trifoliate leaves were harvested every 4 h for 24 h. The mRNA levels of the 10 *GmFT* genes were analyzed by qRT-PCR using gene-specific primers (Supplementary Table 2). In these samples, the expression of *GmFT1b*, *GmFT3a*, *GmFT3b*, and *GmFT5b* transcripts was below detection thresholds (data not shown). The transcript levels of *GmFT2a*, *GmFT2b* and *GmFT5a* were higher in the leaves of floral inductive SD-grown soybean than in those of LD-grown plants (Figure 4). The transcript levels of *GmFT2a*, *GmFT2b*, and *GmFT5a* were highest at 4 h after dawn in SD conditions. *GmFT2a* and *GmFT5a* also exhibited similar diurnal circadian rhythm in LD conditions, even though the relative expression levels were low compared to SD conditions. In contrast, the expression of *GmFT1a* and *GmFT4* were highly induced under floral repressive LD conditions, but their mRNA levels also peaked 4 h after dawn in LD conditions. The results suggested that two subgroups of *GmFT* genes, *GmFT2a*/*GmFT2b*/*GmFT5* and *GmFT1a*/*GmFT4*, might have different roles in day length-dependent flowering in soybean. Interestingly, the mRNA levels of *GmFT6*, which is more closely related to the *GmFT1a*/*GmFT1b*/*GmFT4* subgroup in both sequence homology and in the effect of overexpression in *Arabidopsis* transgenic plants, were higher in SD-grown plants, suggesting that *GmFT6* may have a different mode of action than *GmFT1a*, *GmFT1b*, or *GmFT4* in controlling day length-dependent soybean flowering.

### Exon Swapping Analysis Between *GmFT2a* and *GmFT4*

The effects of overexpression of soybean *FT* genes in *Arabidopsis* transgenic plants and their diurnal expression patterns suggest that *GmFTs* can be divided into two groups based on their biological function. The first group, including *GmFT2a*/*b*, *GmFT3a*/*b*, and *GmFT5a*/*b*, function as floral activators, similar to *Arabidopsis FT*. In contrast, the other group of genes, including *GmFT1a*/*b*, *GmFT4*, and *GmFT6*, likely acquired repressive functions in the soybean flowering process after genome duplication. To map the regions responsible for the antagonistic functions of these two gene subsets, we conducted exon swapping analysis using *GmFT2a* and *GmFT4* genes as representatives of these groups. We generated 10 chimeric genes by exchanging individual exons between *GmFT2a* and *GmFT4*. In addition, the segment B region, which is critical for opposite functions of FT and TFL1 in *Arabidopsis* and for BvFT1 and BvFT2 in beets (Pin et al., 2010), were also exchanged. Each chimeric gene was named using annotations indicating the origin of each of four exons as well as the segment B region; for example, in “*CG2224*,” “CG” indicates chimeric gene, and the numbers indicate that the first three exons are from *GmFT2a*, and the fourth exon from *GmFT4*. The segment B regions from *GmFT2a* and *GmFT4* are indicated as B2 and B4, respectively. The 12 chimeric genes and wild-type forms of *GmFT2a* and *GmFT4* were overexpressed under the control of CaMV 35S promoter in Col-0 plants. Flowering time was analyzed by counting the rosette leaf number of more than 20 independent T1 transformants for each construct.

**FIGURE 5:**
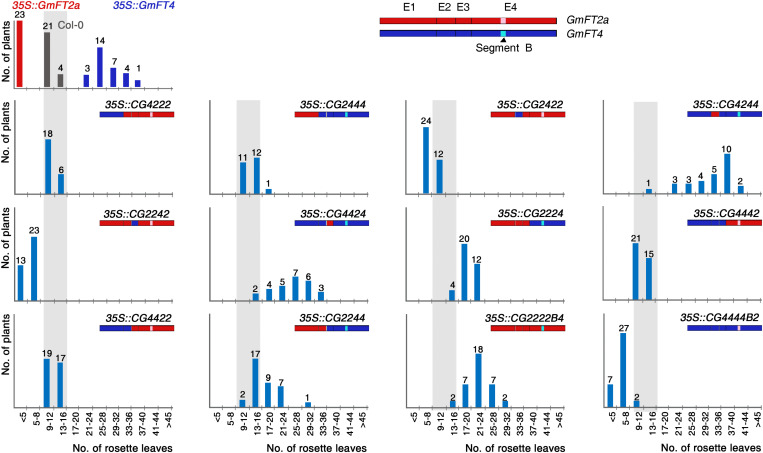
Flowering times of transgenic *Arabidopsis* plants expressing *GmFT2a/GmFT4* exon swapping chimeras. The exons of *GmFT2a* and *GmFT4* are shown as red and blue boxes, respectively. Segment B regions of GmFT2a and GmFT4 are highlighted by pink and cyan colors, respectively. The distribution of flowering times in LD conditions for T1 transformants and control plants (Col-0) are indicated by vertical bars; gray, red, dark blue, and light blue bars for Col-0, 35S::GmFT2a, 35S::GmFT4, and the chimeras, respectively. The number of plants is indicated above each bar.

As previously determined, overexpression of *GmFT2a* and *GmFT4* promoted and delayed flowering in *Arabidopsis*, respectively (Figure 5). Among the four exons in these homologs, swapping of the second, or third exon alone had relatively small effect on the activities of GmFT2a and GmFT4 proteins, slightly reducing the magnitude of the effects of their non-chimeric versions. Most T1 plants expressing *CG2422*, *CG2242*, *CG4244*, and *CG4424* chimeras showed intermediate flowering time between Col-0 and those overexpressing wild-type *GmFT2a* and *GmFT4.* The role of first exon in *GmFT2a* and *GmFT4* was more apparent. Flowering time of *35S::CG4222* and *35S::CG2444* plants was comparable to that of Col-0 plants, indicating that the swapping of the first exon of each gene inactivated both GmFT2a and GmFT4. Similarly, both *35S::CG4422* and *35S::CG2244* plants showed consistent flowering phenotype with Col-0 plants.

As expected, the fourth exon had a stronger effect than other exons on the activities of GmFT2a and GmFT4. *35S::CG2224* plants flowered apparently later than Col-0 plants. The flowering-delaying effects of *GmFT4* in *Arabidopsis* transgenic plants were completely eliminated in *35S::CG4442* plants, even though these plants did not flower as early as *35S::GmFT2a*. A striking phenotypic change in flowering was observed when we overexpressed *CG2222B4* and *CG4444B2* chimeric genes. Although *35S::CG2222B4* plants did not flower as late as *35S:GmFT4*, they did flower much later than Col-0 plants. The most dramatic effects were observed in *35S::CG4444B2* plants; most *35S::CG4444B2* T1 plants flowered earlier than Col-0 plants, and some T1 plants flowered as early as *GmFT2a-*overexpressing plants. Taken together, these results indicated not only that a substitution of the segment B region alone is sufficient to change GmFT2 into a floral repressor and GmFT4 into a floral promoter, but also that the segment B region plays a crucial role in specifying the antagonistic functions of *GmFT2a* and *GmFT4*.

### Identification of the Important Residues in Floral Repressor Function of GmFT4

**FIGURE 6:**
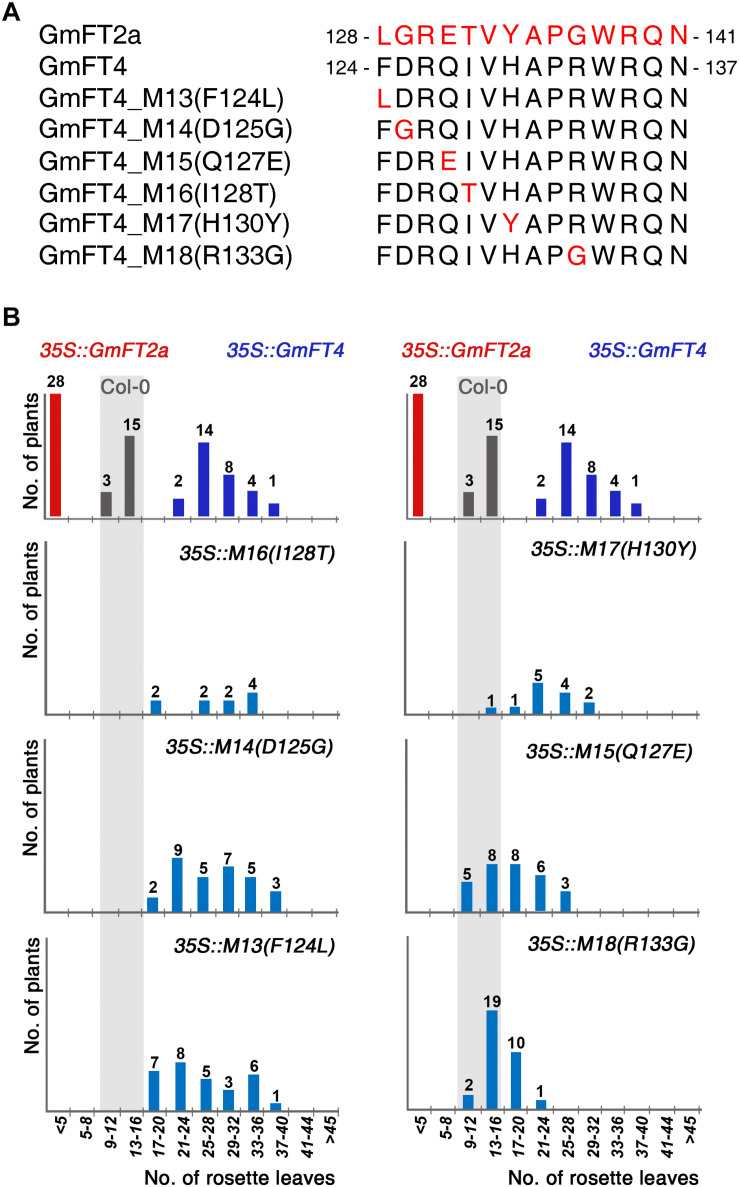
Flowering phenotypes of transgenic *Arabidopsis* plants expressing the GmFT4 segment B-substitution mutants. **(A)** Amino acid sequences of the segment B regions of GmFT2a, GmFT4, and GmFT4 segment B-substitution mutants. The substituted amino acids of GmFT4 with corresponding amino acids of GmFT2a were indicated by red color. **(B)** Flowering times of GmFT4 segment B-substitution mutants. The distribution of flowering times in LD conditions for T1 transformants and control plants (Col-0) are indicated by vertical bars; gray, red, dark blue, and light blue bars for Col-0, 35S::GmFT2a, 35S::GmFT4, and the GmFT4 segment B-substitution mutants, respectively. The number of plants is indicated above each bar.

To identify the critical amino acid residues conferring floral repressor function to GmFT4, we compared amino acid sequences of segment B region between GmFT4 and GmFT2a. Alignment of the 14-amino acid segment B between GmFT4 and GmFT2a showed a difference in 6 amino acids in this region (Figure 6A). To verify the effect of these amino acid substitutions on floral repressor function of GmFT4, we substituted 6 individual amino acids of GmFT4 with corresponding amino acids of GmFT2a and overexpressed them in *Arabidopsis*. Flowering time was again analyzed by counting the rosette leaf number of T1 transformants for each construct. Among the 6 substitution mutants, 4 mutants including 35S::GmFT4 I128T, D125G, F124L, and H130Y showed a similar late-flowering phenotype as 35S::GmFT4 plants. However, two substitutions, Q127E and R133G, strongly suppressed GmFT4 activity. About two-thirds of the T1 transgenic plants overexpressing 35S::GmFT4 R133G showed similar flowering to Col-0 plants (Figure 6B). These results suggest that Arg133 plays an important role in the floral repressor activity of GmFT4.

### Correlation Between Transcript Levels of *GmFT* Genes and Flowering Time of Soybean Accessions

It has been previously shown that expression of the *FT* gene is critical in determining flowering time both in LD and SD plants under proper photoperiod conditions (Kardailsky et al., 1999; Kobayashi et al., 1999; Kojima et al., 2002; Komiya et al., 2008). We therefore investigated the relationship between the expression levels of these 10 *GmFT* homologs and flowering time of soybean accessions. Flowering times of field-grown soybean landraces were determined by counting the number of days from sowing to the date when the first flower was observed in each plant. We selected 24 representative Korean soybean landraces displaying various flowering times and grew them in natural field conditions (Supplementary Table 4). The leaves of soybean landraces were collected before flowering, and the mRNA levels of *GmFT* homologs were analyzed by RT-PCR. Interestingly, among the 10 *GmFT* homologs, transcript levels of *GmFT2a* and *GmFT5a* were higher in early flowering accessions and gradually decreased in later-flowering accessions (Supplementary Figure 3A). In contrast, *GmFT4* mRNA was more abundant in later-flowering accessions than in earlier-flowering ones. The correlation analysis between flowering times of landraces and transcript levels of *GmFT2a*, *GmFT5a*, and *GmFT4* as determined by qRT-PCR indicated significant correlations between expression levels of *GmFT2a*, *GmFT5a*, and *GmFT4* and flowering times of soybean landraces (Supplementary Figure 3B).

**FIGURE 7:**
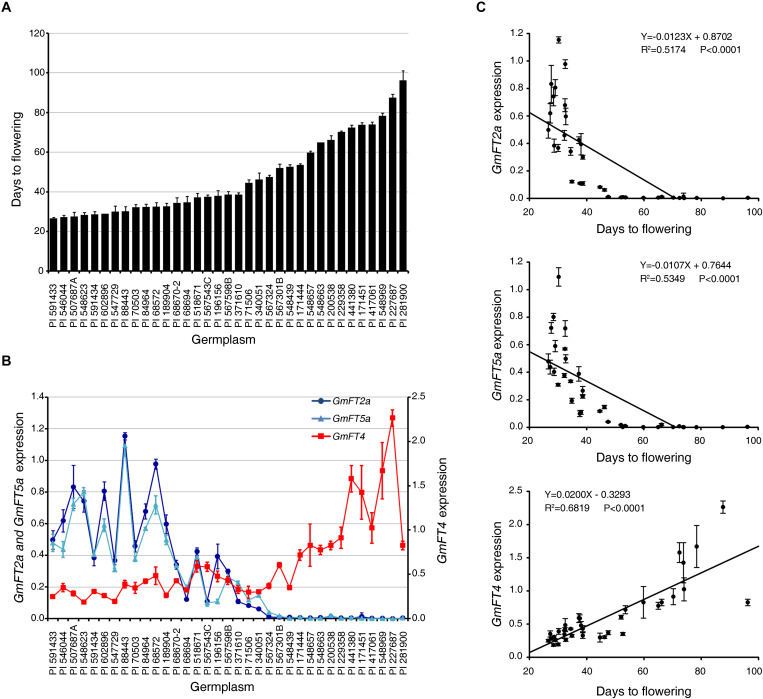
Expression of *GmFT2a*, *GmFT5a*, and *GmFT4* genes in soybean accessions. **(A)** The number of days to flowering of 35 USDA soybean accessions grown in field conditions. **(B)** Evaluation of transcript levels of *GmFT2a*, *GmFT5a*, and *GmFT4* in the third trifoliate (V3) leaves of 30-day old (V4 stage) plants by qRT-PCR with three independent biological replicates. Transcript levels were normalized to *GmPBB2* mRNA levels. **(C)** Correlation analysis between expression levels of *GmFT2a*, *GmFT5a*, and *GmFT4* mRNAs and flowering times of USDA soybean accessions. Data are shown as means ± standard deviation.

To further confirm the relationship between the transcript levels of *GmFT2a*, *GmFT5a*, and *GmFT4* and flowering phenotypes of soybean accessions, we analyzed the expression of these genes by qRT-PCR in the leaves of 35 USDA soybean germplasms exhibiting a broad range of flowering time (Figure 7). Consistently, early flowering accessions displayed higher expression levels of *GmFT2a* and *GmFT5a* transcripts than medium- and late-flowering ones. However, the expression pattern of *GmFT4* in soybean accessions showed the opposite pattern compared to those of *GmFT2a* and *GmFT5a* (Figures 7A,B). Statistical analysis indicated significant correlations between the expression levels of *GmFT2a*, *GmFT5a*, and *GmFT4* and flowering times of USDA soybean germplasms; a negative correlation existed between mRNA levels of *GmFT2a*/*GmFT5a* and the number of days to flowering, but a positive correlation existed for *GmFT4* mRNA levels (Figure 7C). The correlation analysis using various soybean accessions indicated that GmFT2a and GmFT5a might function as floral activators, while GmFT4 might act as a floral repressor, in soybean flowering.

### Seasonal Expression Patterns of *GmFT2a*, *GmFT5a*, and *GmFT4*

**FIGURE 8:**
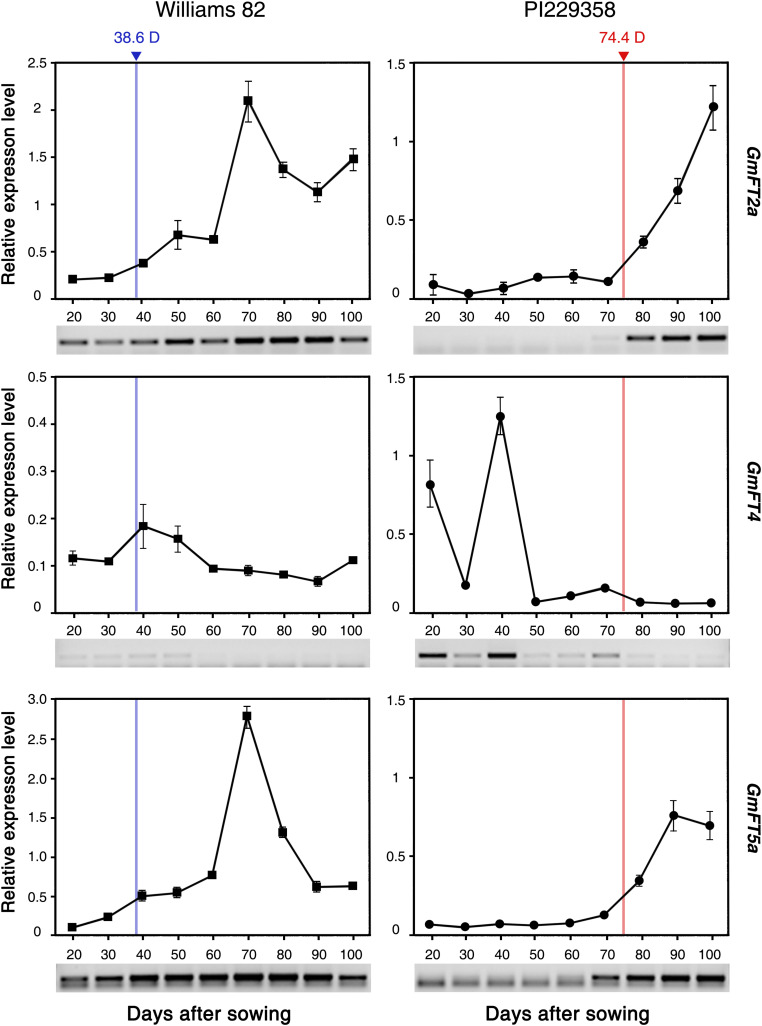
Expression of *GmFT2a*, *GmFT5a*, and *GmFT4* mRNAs in leaves of early (Williams 82)- and late (PI229358)-flowering soybean accessions across different developmental stages. Fully expended trifoliate leaves from the top of the main stem were harvested from three independent plants grown in natural field conditions from 20 to 100 days after sowing. Relative mRNA levels of *GmFT* genes were analyzed by quantitative real-time PCR with three independent biological replicates and normalized to *GmPBB2* mRNA. Days to flowering of Williams 82 (38.6D) and PI229358 (74.4D) are indicated by blue and red line, respectively. The result of independent RT-PCR experiments is also shown below each graph. Data is shown as mean ± standard deviation.

To investigate the correlation between the expression levels of *GmFT2a*, *GmFT5a*, and *GmFT4* mRNAs and seasonal flowering times of soybean accessions, we analyzed their expression patterns in leaves of an early (Williams 82)- and a late (PI229358)-flowering accession during overall growth stages. These seeds were sown in the field and grown in natural conditions. The first flower bloomed at 38.6 and 74.4 days after sowing (DAS) in Williams 82 and PI229358 accessions, respectively. The fully expended trifoliate leaves from the tops of main stems of three independent plants were harvested between 20 and 100 DAS at 10 days intervals. The expression levels of *GmFT2a*, *GmFT5a*, and *GmFT4* were analyzed by qRT-PCR at each time point. In the leaves of early flowering Williams 82 plants, the transcripts of *GmFT2a* and *GmFT5a* were detected at the very early growth stage (20 DAS), and gradually increased during growth and consecutive flowering (Figure 8). Their transcript levels peaked at 70 DAS, and then declined afterward when the new flowers were no longer developing. In the leaves of late-flowering PI229358 plants, the transcripts of *GmFT2a* and *GmFT5a* were not detected during vegetative growth stages; however, their expressions were rapidly induced when PI229358 plants started flowering. In contrast, the expression of *GmFT4* exhibited the opposite pattern to those of *GmFT2a* and *GmFT5a.* Transcripts of *GmFT4* mRNA were barely detected throughout all growth stages of early flowering Williams 82 plants. However, in the leaves of late-flowering PI229358 plants, *GmFT4* was strongly expressed at early vegetative stages (up to 40 DAS), and its expression declined during developmental transition to the reproductive stage. Transcripts of *GmFT4* were not detected after flowering (Figure 8). These results suggested that the accumulation of the *GmFT2a* and *GmFT5a* transcripts in leaves of soybean plants promotes floral induction, but in contrast, high levels of *GmFT4* suppresses floral transition. Furthermore, it also suggests that soybean accessions determine the proper timing of flowering by modulating the cellular levels of floral activators, such as GmFT2a and GmFT5a, and floral suppressors, including GmFT4.

## Discussion

In this study, we identified 17 PEBP family members, including ten *GmFT*, four *GmTFL1*, two *GmBFT*, and one *GmMFT* homolog from soybean. Functional analyses of *GmFT* homologs using overexpression, domain swapping, and amino acid substitutions in *Arabidopsis* transgenic plants indicated that functions of *GmFT* homologs have diversified into two groups: *GmFT2a/b*, *GmFT3a/b*, and *GmFT5a/b* function as floral promoters; in contrast, *GmFT1a/b*, *GmFT4*, and *GmFT6* function as floral repressors. Expression analyses of *GmFT* genes in soybean accessions exhibiting various flowering times suggested that the relative expression level between floral promoters *GmFT2a/GmFT5a* and floral repressor *GmFT4* is one of the critical factors in determining flowering time in response to environmental changes. Our results suggest that soybean plants determine the optimum flowering time during growing seasons by modulating the relative cellular levels of floral activators and repressors GmFT homologs, and that this modulation may also be important for the adaptation of soybeans to their habitats.

### Functional Diversification of Soybean *FT* Homologs in Control of Flowering Time

Since the first identification of the *FT* gene in *Arabidopsis thaliana*, biological functions of *FT* homologs as floral activators have been widely verified in various plant species (Kardailsky et al., 1999; Kobayashi et al., 1999; Abe et al., 2005; Wigge et al., 2005; Wickland and Hanzawa, 2015). However, recently, *FT* homologs exhibiting opposite functions to *Arabidopsis FT* have been reported from other plant species, especially in crops, including sunflower, sugar beet, onion, tobacco, sugarcane, longan, and soybean (Blackman et al., 2010; Harig et al., 2012; Lee et al., 2013; Winterhagen et al., 2013; Coelho et al., 2014; Zhai et al., 2014; Liu et al., 2018). These results suggest that the functions of *FT* homologs have diverged through neo- or sub-functionalization, and during evolution acquired a repressive function in flowering. Moreover, some repressor *FT* homologs have been selected for during domestication and breeding (Wang et al., 2015; Jiang et al., 2019). In this study, we characterized the functions of 10 *GmFT* homologs in flowering by overexpressing them in *Arabidopsis*. Overexpression of six *GmFT*s, *GmFT2a/b*, *GmFT3a/b*, and *GmFT5a/b*, promoted flowering. Among these, *GmFT3a* showed a relatively milder effect on flowering than the others. In contrast, transgenic *Arabidopsis* plants overexpressing *GmFT1a/b*, *GmFT4*, and *GmFT6* showed significantly delayed flowering times compared to WT plants (Table 1 and Figure 3). *GmFT4* exhibited the strongest floral repressor activity as indicated by the number of rosette leaves. Interestingly, while *35S::GmFT6* plants produced fewer rosette leaves than *35S::GmFT4* plants prior to bolting, *35S::GmFT6* plants produced the highest number of cauline leaves among the 10 *GmFT* homologs (Table 1). This result suggests that *GmFT6* has a different mechanism of action in floral repression than the other floral inhibitors, *GmFT1a/b* and *GmFT4*. Consistently, in contrast to *GmFT1a* and *GmFT4*, mRNA levels of *GmFT6* were higher in floral inductive SD-grown soybean leaves than in LD-grown plants, which is a typical expression pattern of floral activator *GmFT* homologs *GmFT2a/b* and *GmFT5a* (Figure 4). Moreover, gene expression patterns of *GmFT4* and *GmFT6* were complementary to each other. The mRNA level of *GmFT4* was highest in newly developing young leaves (TI4 leaves of V4 and R2 stages), and gradually decreased in older leaves (TI3, TI 2, and TI1 leaves); however, mRNA levels of *GmFT6* showed the opposite pattern, wherein they were lowest in TI4 and highest in TI1 leaves (Figure 2). This complementary expression pattern was also observed in the analysis of seasonal expression patterns of *GmFT*s. *GmFT4* was predominantly expressed in the vegetative stage of soybean accessions, but its expression was suppressed by flowering (Figure 8). However, transcripts of *GmFT6* began to increase after flowering when *GmFT4* transcripts were declining (Supplementary Figure 4). Taken together, these results suggest that biological function of GmFT6 protein has diverged to become a floral repressor, similar to GmFT1a and GmFT4; however, its gene expression pattern is closer to that of floral activators *GmFT2a* and *GmFT5a*. Future studies are required to characterize in more detail the role of GmFT6 in soybean flowering.

### Amino Acids Specifying the Antagonistic Functions of GmFT Homologs

Among *Arabidopsis* PEBP family members, FT and TFL1 exhibit opposite functions in flowering, and two critical amino acids that play a decisive role in determining these opposite functions have been identified: Tyr85 and Gln140 in FT versus His88 and Asp144 in TFL1 (Hanzawa et al., 2005; Ahn et al., 2006). The analysis of crystal structures of FT and TFL1 suggests that these amino acid pairs are located at the entrance to ligand-binding pockets, where partner proteins possibly interact with FT/TFL1, and different interaction patterns between Tyr85-Gln140 in FT and His88-Asp144 in TFL1 may contribute to their opposite functions (Ahn et al., 2006). Two critical amino acids in specifying FT function, Tyr85 and Gln140, are also conserved in GmFT homologs, excepting only GmFT5a/b (Figure 1C), indicating that these residues are not critical in determining the repressive functions of GmFT homologs. To identify the critical amino acid(s) specifying these antagonistic functions of GmFT homologs, we conducted exon swapping and amino acid substitution analyses using GmFT2a and GmFT4 as representatives of floral activators and repressors, respectively. The exon swapping experiment indicated that the segment B region in the fourth exon, which is known to be critical for FT versus TFL1 function (Ahn et al., 2006) and which has been identified as critical for opposite functions of beet FT homologs (Pin et al., 2010), is also important in the opposite functions of GmFT2a and GmFT4 (Figure 5). To pinpoint the decisive residue(s) in the segment B region, we substituted 6 individual amino acids in this region of GmFT4 with the corresponding residues of GmFT2a, and analyzed their respective effects on GmFT4 repressive activity. Among them, substitution of Arg133 of GmFT4 with Gly present in GmFT2a exhibited the strongest effect on suppression of GmFT4 activity (Figure 6). However, the R133G substitution was not sufficient to change GmFT4 function to that of a floral activator such as GmFT2a. These results suggest that the Arg133 residue is important and necessary for the floral repressor GmFT4 activity; however, to convert GmFT4 into a floral activator, other amino acid changes might be additionally required.

Previously, extensive random mutagenesis assays of *Arabidopsis* FT successfully identified critical residues that are sufficient to convert FT into TFL1-like protein, including Glu109, Trp138, Gln140, and Asn152 (Ho and Weigel, 2014). Moreover, two aromatic residues, Tyr134 and Trp138, were proposed as critical amino acids for FT function. Consistently, most plant FT homologs exhibiting repressor activity, such as BvFT1, AcFT4, HaFT1, ScFT1, and NtFTs, contain non-tyrosine and non-tryptophan amino acids at these sites (Wickland and Hanzawa, 2015). However, this is not the case with GmFT homologs. All GmFT homologs identified here possess Trp residues at the position corresponding to Trp138 of AtFT. In addition, at the corresponding position of Tyr134, floral activators GmFT5a/b contain Ile residues instead of Tyr, and floral repressor GmFT1b contains Tyr (Figure 1C). Moreover, substitution of His130 of GmFT4 to the corresponding Tyr residue of GmFT2a had a weak effect on GmFT4 repressor activity (Figure 6). These results suggest that soybean FT homologs have acquired diverse functions during evolution compared to the FT homologs in other plants.

### Expressional Diversification of *GmFT* Homologs in Soybean Accessions

Soybean, a SD plant, originated in East Asia and was mainly cultivated in high latitudes. Soybean cultivars grown in these regions are often photoperiod-insensitive and exhibit a fast life cycle, including early flowering, to successfully produce seeds during short growing season. However, cultivation of soybeans was extended to lower latitudes after the identification of soybean accessions exhibiting the long juvenile period trait of delayed flowering under SD conditions (Sinclair et al., 2005; Lu et al., 2017). Identification of genetic variation in many of flowering and maturity genes has mainly contributed to broadening of the region of soybean adaptability and cultivation (Watanabe et al., 2012). Here, we suggest that functional diversification of GmFT homologs contributes to adaptation of soybean accessions to diverse environments. In addition, diversification in gene expression patterns of *GmFT* homologs also plays an important role in adaptation and domestication of soybean cultivars. Our results showed that early flowering soybean accessions exhibited high expression levels of floral activators *GmFT2a* and *GmFT5a*, however, their expressions were strongly suppressed during the vegetative stages (V4) of late-flowering accessions. In contrast, floral repressor *GmFT4* showed the exact opposite expression pattern (Figure 7 and Supplementary Figure 2). Consistently, during the juvenile period of late-flowering accessions, while the expression of *GmFT2a* and *GmFT5a* was low levels, *GmFT4* was highly expressed. However, the transcription of *GmFT2a* and *GmFT5a* was induced along with flowering (Figure 8). These results suggest that *GmFT* homologs acting as floral repressors, such as *GmFT4*, suppress flowering until the proper timing of flowering. Once the environment becomes suitable for flowering, soybean turns on the transcription of floral activators *GmFT2a* and *GmFT5a* to initiate flowering.

## Conclusion

Taken together, we conclude that not only the existence of various *GmFT* homologs with antagonistic functions, but also the differential regulation of their gene expressions are critical for the adaptation of soybean accessions to diverse habitats and for maximizing yields.

## Data Availability Statement

The original contributions presented in the study are included in the article/Supplementary Material, further inquiries can be directed to the corresponding author/s.

## Author Contributions

SHL, CWC, KMP, and MCK designed and performed the experiments, analyzed data, and wrote the manuscript. W-HJ, HJC, DB, HMC, BJJ, MSP, DHN, and LHL performed experiments. SIS, JIC, and MCK discussed and commented on results, and revised the manuscript. All authors approved the final manuscript.

## Conflict of Interest

The authors declare that the research was conducted in the absence of any commercial or financial relationships that could be construed as a potential conflict of interest.

## Footnotes

1 http://www.mbio.ncsu.edu/bioedit

2 https://www.r-project.org/

3 http://www.phytozome.net/soybean

## Supplementary Material

Supplementary Figure 1 Amino acid sequence alignment of the soybean and *Arabidopsis* PEBP family members.

Supplementary Figure 2 Expression of *GmTFL1* homologs in flowers.

Supplementary Figure 3 mRNA expression of *GmFT* genes in soybean accessions.

Supplementary Figure 4 *GmFT6* mRNA expression in leaves of early (Williams 82) and late (PI229358) flowering soybean accessions across different developmental stages.

Supplementary Table 1 Primers used for full-length cDNA cloning.

Supplementary Table 2 Primers used for RT-PCR and quantitative real-time PCR.

Supplementary Table 3 Flowering times of *Arabidopsis* transgenic T1 lines overexpressing *GmFT* homologs.

Supplementary Table 4 Flowering times of soybean landraces determined by the number of days to flowering.

Supplementary Table 5 Primers used for exon swapping.

Supplementary Table 6 Primers used for amino acid substitution.
