# Supplementary material for: Diversification in Functions and Expressions of Soybean FLOWERING LOCUS T Genes Fine-Tunes Seasonal Flowering
Source: Front Plant Sci. 2021 Apr 26;12:613675. doi: 10.3389/fpls.2021.613675 (PMC8107440; doi:10.3389/fpls.2021.613675)
Supplement: Supplementary Figure 1 — Amino acid sequence alignment of the soybean and Arabidopsis PEBP family members. [file Data_Sheet_1.docx]

***Supplementary Material***

**Diversification in Functions and Expressions of Soybean *FLOWERING LOCUS T* Genes Fine-tunes Seasonal Flowering**

Su Hyeon Lee, Cheol Woo Choi, Kyoung Mi Park, Wook-Hun Jung, Hyun Jin Chun, Dongwon Baek, Hyun Min Cho, Byung Jun Jin, Mi Suk Park, Dong Hyeon No, Lack Hyeon Lim, Sang In Shim, Jong Il Chung, and Min Chul Kim**^*^**

*** Corresponding author:**

Dr. Min Chul Kim

Division of Applied Life Science, Gyeongsang National University, Jinju 52828, Korea

Tel: +82-55-772-1874

Fax: +82-55-772-1879

E-mail: [mckim@gnu.ac.kr](mailto:mckim@gnu.ac.kr)

ORCID IDs: 0000-0001-5472-992X (M.C.K.).


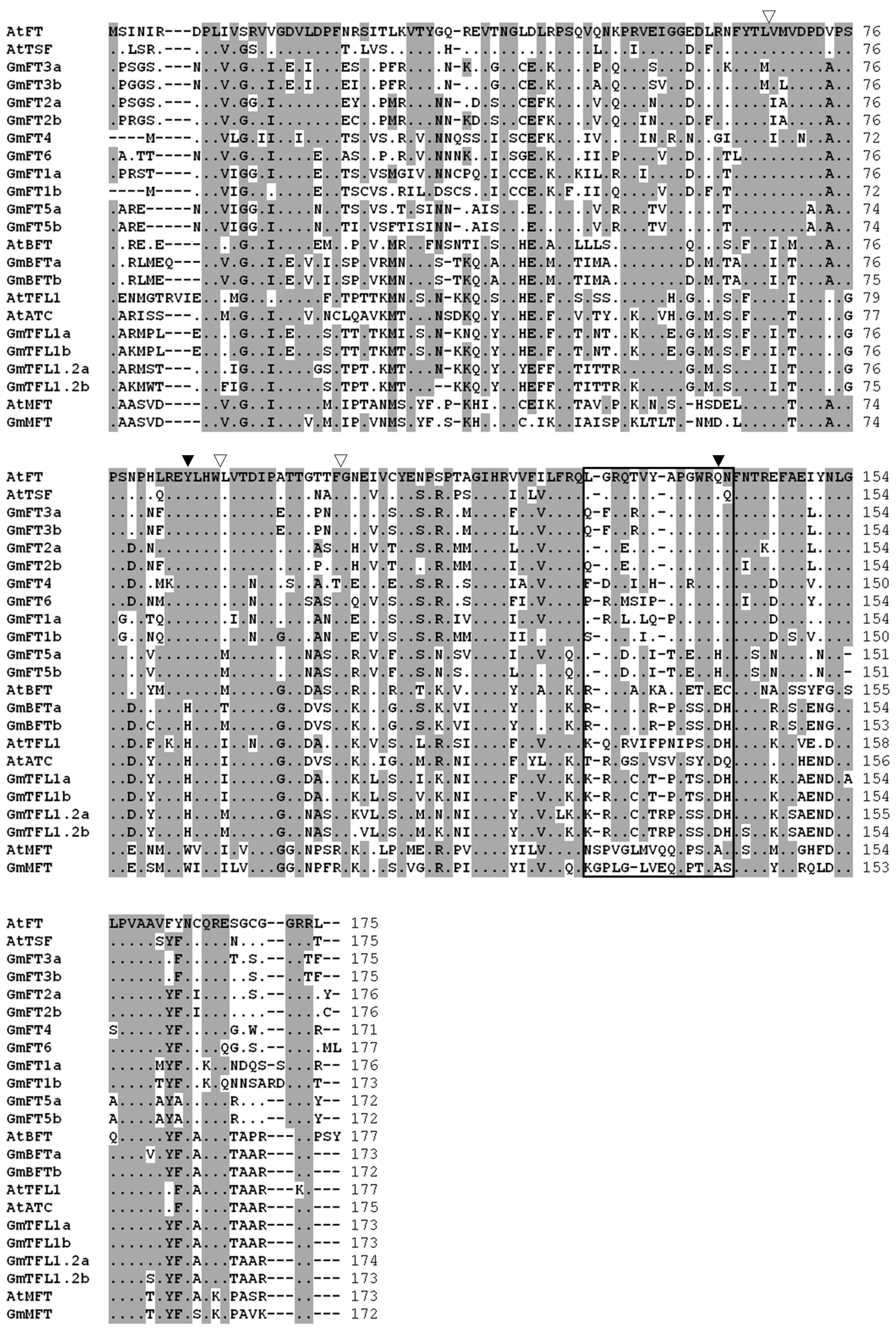


**Supplementary Figure S1.** Amino acid sequence alignment of the soybean and *Arabidopsis* PEBP family members. Black triangles on the upper row indicate the Tyr85(Y)/His88(H) and Gln140(Q)/Asp144(D) residues corresponding to the antagonistic function of *Arabidopsis* FT and TFL1 in flowering. The exon boundaries of AtFT are indicated by white triangles. The conserved segmental region B at exon 4, composed of 14 amino acids, is boxed. Amino acids identical to those of *Arabidopsis* FT are indicated by dots and gray boxes.


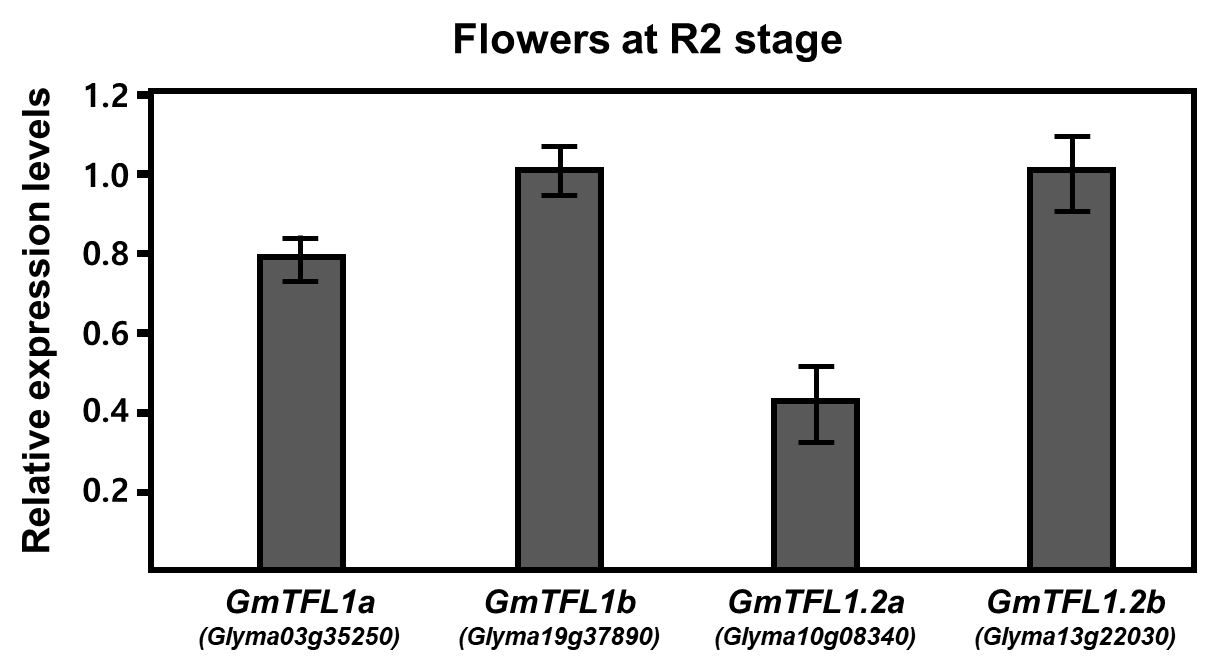


**Supplementary Figure S2.** Expression of *GmTFL1* homologs in flowers. Flowers were harvested from soybean plants grown in natural green house conditions at R2 stage. Relative mRNA levels of *GmTFL1* homologs were analyzed by quantitative real-time PCR and normalized to *GmPBB2*. Data is shown as mean ± standard deviation.


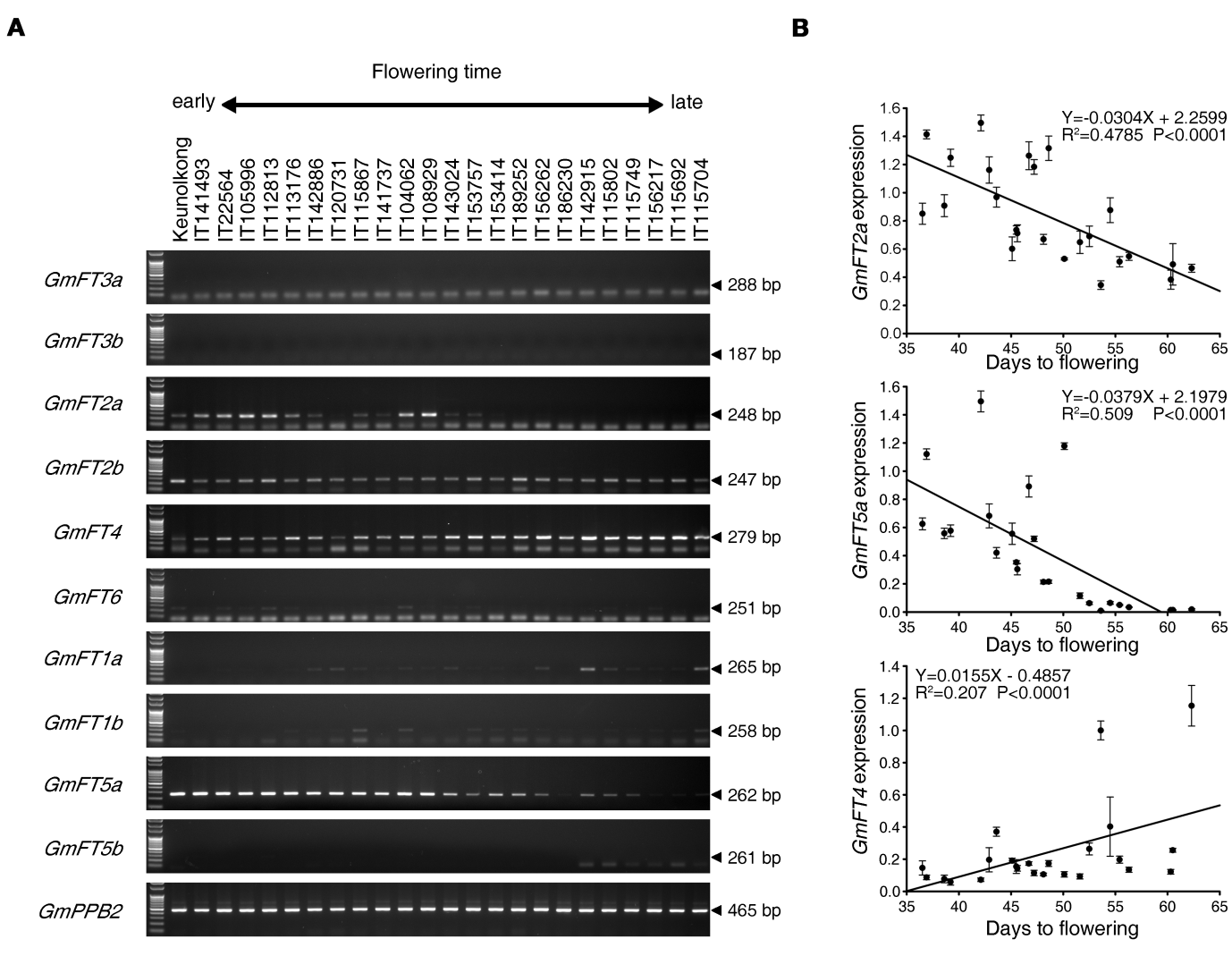


**Supplementary Figure S3.** mRNA expression of *GmFT* genes in soybean accessions. (A) The expression levels of *GmFT* genes were analyzed by RT-PCR. Total RNAs were extracted from the third trifoliate (V3) leaves of 30-day old (V4 stage) field-grown soybean accessions, which exhibited various flowering phenotypes. Gene expression was normalized to *GmPBB2* mRNA levels. The expected sizes of the PCR products of each gene are indicated. (B) Correlation analysis between expression levels of *GmFT2a*, *GmFT5a*, and *GmFT4* genes and flowering times of soybean accessions. Data is shown as mean ± standard deviation.


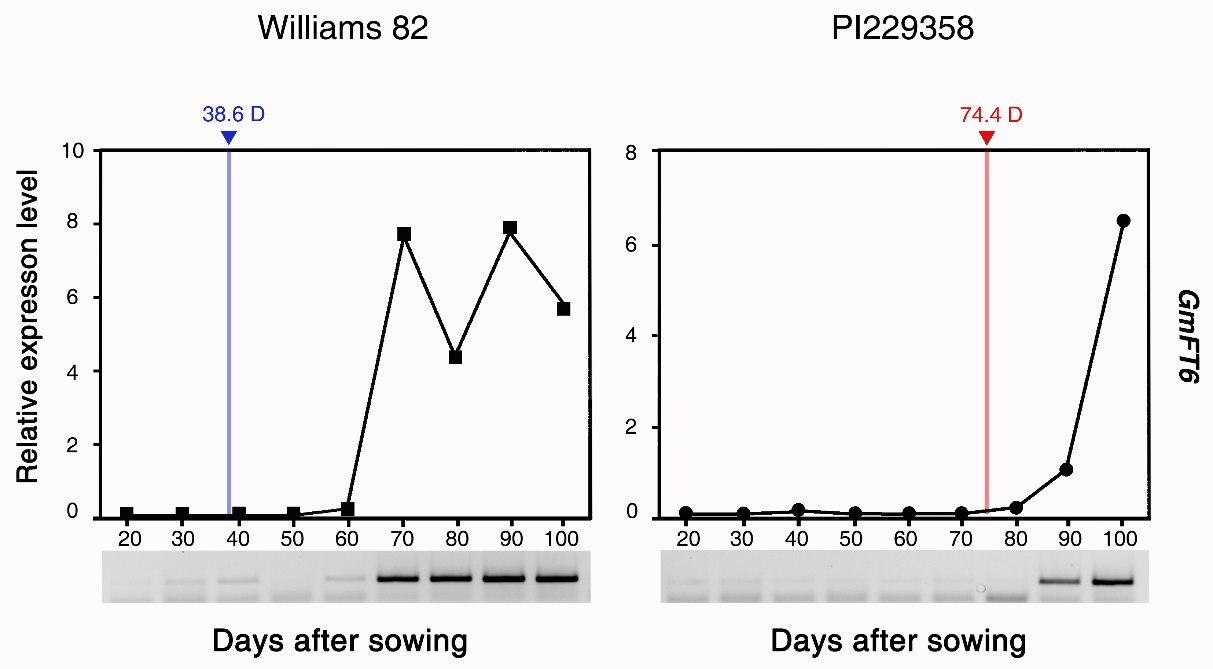


**Supplementary Figure S4.** *GmFT6* mRNA expression in leaves of early (Williams 82) and late (PI229358) flowering soybean accessions across different developmental stages. Fully expanded trifoliate leaves from the top of main stem were harvested from three independent plants grown in natural field conditions from 20 to 100 days after sowing. Relative mRNA levels of *GmFT6* were analyzed by quantitative real-time PCR and normalized to *GmPBB2*. The number of days until flowering of Williams 82 (38.6D) and PI229358 (74.4D) are indicated by blue and red line, respectively. The result of independent RT-PCR experiments is also shown below each graph. Data is shown as mean ± standard deviation.

**Supplementary Table S1.** Primers used for full-length cDNA cloning.

| Gene | Primer name^*^ | Primer sequence^**^ |
| --- | --- | --- |
| *GmFT1a*  *GmFT1b*  *GmFT2a*  *GmFT2b*  *GmFT3a*  *GmFT3b*  *GmFT4*  *GmFT5a*  *GmFT5b*  *GmFT6*  *GmTFL1a*  *GmTFL1b*  *GmTFL1.2a*  *GmTFL1.2b*  *GmBFTa*  *GmBFTb*  *GmMFT* | MG-0890 (F)  MG-0861 (R)  MG-1146 (F)  MG-1147 (R)  MG-0982 (F)  MG-0984 (R)  MG-1189 (F)  MG-1116 (R)  MG-0878 (F)  MG-0879 (R)  MG-1188 (F)  MG-1114 (R)  MG-1150 (F)  MG-1151 (R)  MG-1190 (F)  MG-1128 (R)  MG-1197 (F)  MG-1129 (R)  MG-1148 (F)  MG-1149 (R)  MG-0618 (F)  MG-0619 (R)  MG-1192 (F)  MG-1105 (R)  MG-1193 (F)  MG-1107 (R)  MG-1194 (F)  MG-1108 (R)  MG-0620 (F)  MG-0621 (R)  MG-1191 (F)  MG-1111 (R)  MG-0616 (F)  MG-0617 (R) | ATGCCTAGATCAACGGACCCTCTTG  TTATCTTCTTCTTCCACTGCTTTGATC  CATGGACCCTCTTGTCATTGGACGTG  GCTCATGTCCTTCTTCCATCTCTTGCG  ATGCCTAGTGGAAGTAGGGATCC  TTAGTATAACCTCCTTCCACCAG  AATGCCTCGTGGAAGTAGGGAC  CACATTCAAAAGTCCCTTATTTTATAAAGATC  TTATGCCTAGTGGTAGTAGGAACCC  ATTCAAAATGTTCTACCACCAGAGCC  TATGCCTGGCGGTAGTAGGAAC  ACTTTTGGTACATTAACAGAAGAGATAGC  GATGGACCCCCTTGTTCTTGGACG  GATCATCTCCTTCGTCCACCCCAAC  TATGGCACGGGAGAACCCTCTTG  GCTGGAGTAAGGCATCCAAGAATCTTC  TATGGCACGGGAGAACCCTCTTG  GCTAGAGTAGAGGCATCCAAAAAAATCTTC  CAATGGCTATTACAACGAACCCTCTTG  GTTATAACATTAGCCTCCTTCCCCCAG  ATGGCAAGAATGCCTTTAGAGCCTC  CTAGCGTCTTCTTGCAGCCGTTTC  TATGGCAAAAATGCCTTTAGAGCC  GGCAAAACCAGCAGCTACTTAGC  TATGGCAAGGATGTCGACAGATC  GCTAGATATAATAGCTACTAGTGCAATAAGC  TATGGCAAAGATGTGGACAGATC  CTTGCATATATTGCACTAGTAACTATAACTATAAC  ATGTCTAGGCTAATGGAACAACCAC  TCACCTCCTTCTTGCAGCAGTCTC  AATGTCTAGGCTCATGGAACCAC  GATTTAATCAAACACACTTATCCTTATTGT  ATGGCAGCCTCCGTGGATCCCC  TCAGCGCCTCTTAACAGCAGGTTC |

^*^F and R in parentheses indicate forward and reverse, respectively.

^**^Primer sequence is written from 5’ to 3’.

**Supplementary Table S2.** Primers used for RT-PCR and quantitative real-time PCR.

| Gene | Primer name^*^ | Primer sequence^**^ |
| --- | --- | --- |
| *GmFT1a*  *GmFT1b*  *GmFT2a*  *GmFT2b*  *GmFT3a*  *GmFT3b*  *GmFT4*  *GmFT5a*  *GmFT5b*  *GmFT6*  *GmTFL1a*  *GmTFL1b*  *GmTFL1.2a*  *GmTFL1.2b*  *GmBFTa*  *GmBFTb*  *GmMFT*  *GmPBB2* | MG-1123 (F)  MG-1124 (R)  MG-1125 (F)  MG-1126 (R)  MG-1117 (F)  MG-1118 (R)  MG-1115 (F)  MG-1116 (R)  MG-1112 (F)  MG-1113 (R)  MG-1112 (F)  MG-1114 (R)  MG-1119 (F)  MG-1120 (R)  MG-1508 (F)  MG-1509 (R)  MG-1197 (F)  MG-1129 (R)  MG-1148 (F)  MG-1149 (R)  MG-1419 (F)  MG-1420 (R)  MG-1103 (F)  MG-1105 (R)  MG-1106 (F)  MG-1107 (R)  MG-1106 (F)  MG-1108 (R)  MG-2227 (F)  MG-2228 (R)  MG-1191 (F)  MG-1111 (R)  MG-1101 (F)  MG-1102 (R)  MG-1097 (F)  MG-1098 (R) | CCTATGAAAGTCCACGTCCAATAGTAG  CATGTTGAGCTGATAAAAACTCGGG  GGTAGACAAACTATATATGCTCCAGGATGG  CAACAATTTGAGTGTCTAGCTCCCGAG  ATGGGGATTCATCGTTTGGTG  TGAAACCCTCAACGAAGCTTATACTAC  ATGATGGGGATCCATCGTATAGTC  CACATTCAAAAGTCCCTTATTTTATAAAGATC  TTCCGTCAACAGTTTAGACAGAGG  TCGGTTACATATTCATGTGTATGTGAC  TTCCGTCAACAGTTTAGACAGAGG  ACTTTTGGTACATTAACAGAAGAGATAGC  CCAACTTCTGGGATTCATCGGATTGC  ACAACGTGCGCAGTGTGACTGCAC  ACCCTTCAGTAGGGATTCATCGAATC  CTGGAGTAAGGCATCCAAGAATCTTC  TATGGCACGGGAGAACCCTCTTG  GCTAGAGTAGAGGCATCCAAAAAAATCTTC  CAATGGCTATTACAACGAACCCTCTTG  GTTATAACATTAGCCTCCTTCCCCCAG  AAGAAGACGCTAGCTATAGCTGCTG  GTAAAATAAAACGTAGCATACACACGG  TTGGGAAAGAGTTGGTGAGCTATG  GGCAAAACCAGCAGCTACTTAGC  CCCAAATGAGGCATTTGTTGT  GCTAGATATAATAGCTACTAGTGCAATAAGC  CCCAAATGAGGCATTTGTTGT  CTTGCATATATTGCACTAGTAACTATAACTATAAC  ATCTCGAGATGTCTAGGCTAATGGAACAACCA  ATTCTAGATCACCTCCTTCTTGCGGCA  AATGTCTAGGCTCATGGAACCAC  GATTTAATCAAACACACTTATCCTTATTGT  TGGAGCAACCACCAACTCGAGCAAG  CCATGCATATCTCAAGAGAGACACTGATGG  TGCCGAAGAAACGCAATGCTTCAA  TGCAGCAAGTGAACCTGATCCCAT |

^*^F and R in parentheses indicate forward and reverse, respectively.

^**^Primer sequence is written from 5’ to 3’.

**Supplementary Table S3.** Flowering times of *Arabidopsis* transgenic T1 lines overexpressing *GmFT* homologs.

| **Name** |  | **1** | **2** | **3** | **4** | **5** | **6** | **7** | **8** | **9** | **10** | **11** | **12** | **13** | **14** | **15** | **16** | **17** | **18** | **19** | **20** | **21** | **22** | **23** | **24** | **25** | **26** | **27** | **28** | **29** | **30** | **31** | **32** | **33** | **34** | **35** | **36** | **37** | **Ave.** | **STD** | **n.** |
| --- | --- | --- | --- | --- | --- | --- | --- | --- | --- | --- | --- | --- | --- | --- | --- | --- | --- | --- | --- | --- | --- | --- | --- | --- | --- | --- | --- | --- | --- | --- | --- | --- | --- | --- | --- | --- | --- | --- | --- | --- | --- |
| **Wild-type**  **(Col-0)** |  | **Non-transgenic line** | | | | | | | | | | | | | | | | | | | |  |  |  |  |  |  |  |  |  |  |  |  |  |  |  |  |  |  |  |  |
|  | **CLN** | **3** | **3** | **2** | **2** | **2** | **3** | **2** | **2** | **2** | **3** | **3** | **3** | **3** | **3** | **3** | **2** | **3** | **3** | **2** | **3** |  |  |  |  |  |  |  |  |  |  |  |  |  |  |  |  |  | **2.6** | **0.50** | **20** |
|  | **RLN** | **11** | **12** | **12** | **11** | **11** | **12** | **11** | **12** | **10** | **12** | **12** | **11** | **11** | **10** | **9** | **9** | **9** | **10** | **9** | **9** |  |  |  |  |  |  |  |  |  |  |  |  |  |  |  |  |  | **10.7** | **1.18** |  |
| ***35S:GmFT2a*** |  | **T1 #1** | | | | | | | | | **T1 #3** | | | | | | | **T1 #4** | | | **T1 #9** | | | |  |  |  |  |  |  |  |  |  |  |  |  |  |  |  |  |  |
|  | **CLN** | **2** | **2** | **2** | **2** | **2** | **3** | **2** | **2** | **2** | **2** | **2** | **3** | **2** | **3** | **2** | **3** | **2** | **3** | **2** | **2** | **2** | **2** | **2** |  |  |  |  |  |  |  |  |  |  |  |  |  |  | **2.2** | **0.42** | **23** |
|  | **RLN** | **3** | **4** | **4** | **4** | **4** | **4** | **4** | **3** | **3** | **3** | **3** | **4** | **4** | **3** | **3** | **3** | **3** | **3** | **3** | **3** | **3** | **3** | **3** |  |  |  |  |  |  |  |  |  |  |  |  |  |  | **3.3** | **0.49** |  |
| ***35S:GmFT2b*** |  | **T1 #6** | | | | | | | | | **T1 #10** | | | | | | **T1 #12** | | | | | | | | | | | **T1 #15** | | | | | | | | | | |  |  |  |
|  | **CLN** | **2** | **2** | **1** | **2** | **2** | **2** | **2** | **2** | **2** | **2** | **2** | **2** | **2** | **3** | **2** | **2** | **2** | **2** | **2** | **2** | **2** | **2** | **2** | **2** | **2** | **2** | **1** | **2** | **2** | **2** | **2** | **2** | **2** | **2** | **2** | **1** | **2** | **1.9** | **0.33** | **37** |
|  | **RLN** | **3** | **3** | **2** | **3** | **3** | **3** | **3** | **3** | **3** | **3** | **3** | **3** | **3** | **3** | **3** | **3** | **3** | **3** | **3** | **3** | **3** | **3** | **3** | **3** | **3** | **3** | **2** | **3** | **3** | **3** | **3** | **3** | **3** | **3** | **3** | **2** | **3** | **2.9** | **0.28** |  |
| ***35S:GmFT3a*** |  | **T1 #2** | | | | | | | | | **T1 #4** | | | | | | | | **T1 #11** | | | **T1 #13** | | | | | | | |  |  |  |  |  |  |  |  |  |  |  |  |
|  | **CLN** | **4** | **4** | **4** | **4** | **3** | **4** | **4** | **2** | **4** | **3** | **3** | **3** | **2** | **3** | **4** | **4** | **3** | **3** | **3** | **3** | **5** | **4** | **2** | **3** | **2** | **2** | **2** | **2** |  |  |  |  |  |  |  |  |  | **3.2** | **0.86** | **28** |
|  | **RLN** | **6** | **6** | **5** | **8** | **6** | **7** | **6** | **4** | **7** | **5** | **4** | **6** | **4** | **5** | **7** | **7** | **7** | **7** | **4** | **3** | **9** | **6** | **4** | **5** | **5** | **4** | **5** | **7** |  |  |  |  |  |  |  |  |  | **5.7** | **1.44** |  |
| ***35S:GmFT3b*** |  | **T1 #5** | | | | | | | | | **T1 #6** | | | | | | | | | **T1 #8** | | | | | | | | **T1 #9** | | | | | | |  |  |  |  |  |  |  |
|  | **CLN** | **2** | **3** | **2** | **2** | **2** | **1** | **1** | **2** | **2** | **2** | **2** | **1** | **5** | **2** | **3** | **2** | **2** | **3** | **3** | **3** | **2** | **3** | **2** | **2** | **1** | **2** | **2** | **1** | **2** | **2** | **2** | **2** | **2** |  |  |  |  | **2.1** | **0.78** | **33** |
|  | **RLN** | **3** | **3** | **3** | **3** | **3** | **3** | **3** | **3** | **3** | **3** | **3** | **3** | **3** | **3** | **3** | **3** | **3** | **4** | **3** | **3** | **3** | **3** | **3** | **3** | **3** | **3** | **3** | **3** | **3** | **3** | **3** | **3** | **3** |  |  |  |  | **3.0** | **0.17** |  |
| ***35S:GmFT5a*** |  | **T1 #9** | | | | | | | | | **T1 #12** | | | | | | | | | **T1 #13** | | | | | | | | | **T1 #19** | | | | | | | |  |  |  |  |  |
|  | **CLN** | **2** | **1** | **2** | **2** | **1** | **3** | **1** | **2** | **3** | **2** | **2** | **2** | **3** | **2** | **3** | **1** | **2** | **1** | **2** | **2** | **1** | **1** | **2** | **3** | **1** | **2** | **3** | **1** | **1** | **1** | **1** | **2** | **1** | **2** | **2** |  |  | **1.8** | **0.72** | **35** |
|  | **RLN** | **3** | **3** | **3** | **3** | **3** | **4** | **3** | **3** | **3** | **3** | **3** | **3** | **3** | **3** | **3** | **3** | **3** | **3** | **4** | **3** | **2** | **2** | **3** | **3** | **3** | **3** | **3** | **3** | **3** | **3** | **2** | **3** | **2** | **3** | **3** |  |  | **2.9** | **0.42** |  |
| ***35S:GmFT5b*** |  | **T1 #4** | | | | | | | | | **T1 #10** | | | | | | | | | **T1 #11** | | | | | | | | | **T1 #12** | | | | | | | |  |  |  |  |  |
|  | **CLN** | **1** | **1** | **1** | **1** | **1** | **1** | **1** | **1** | **1** | **2** | **1** | **1** | **2** | **2** | **2** | **2** | **2** | **2** | **2** | **3** | **2** | **2** | **1** | **1** | **2** | **1** | **2** | **2** | **1** | **1** | **2** | **2** | **2** | **2** | **2** |  |  | **1.6** | **0.56** | **35** |
|  | **RLN** | **3** | **3** | **3** | **4** | **3** | **4** | **3** | **3** | **3** | **3** | **3** | **3** | **3** | **3** | **3** | **3** | **3** | **3** | **3** | **3** | **3** | **3** | **4** | **2** | **3** | **2** | **3** | **3** | **5** | **3** | **4** | **3** | **3** | **3** | **3** |  |  | **3.1** | **0.53** |  |
| ***35S:GmFT1a*** |  | **T1 #4** | | | | | | | | | **T1 #10** | | | | | | | | **T1 #25** | | | | | | | | | **T1 #28** | | | | | | | | |  |  |  |  |  |
|  | **CLN** | **4** | **3** | **3** | **3** | **5** | **5** | **5** | **5** | **3** | **5** | **5** | **8** | **4** | **5** | **5** | **4** | **5** | **4** | **4** | **5** | **4** | **6** | **5** | **5** | **3** | **5** | **5** | **5** | **7** | **4** | **4** | **5** | **5** | **3** | **3** |  |  | **4.5** | **1.12** | **35** |
|  | **RLN** | **17** | **17** | **15** | **13** | **16** | **20** | **21** | **19** | **15** | **21** | **19** | **23** | **24** | **18** | **16** | **17** | **18** | **15** | **17** | **18** | **18** | **21** | **18** | **23** | **15** | **17** | **17** | **22** | **23** | **19** | **15** | **21** | **20** | **15** | **15** |  |  | **18.2** | **2.84** |  |
| ***35S:GmFT1b*** |  | **T1 #4** | | | | | | | | | **T1 #8** | | | | | | | | **T1 #12** | | | | | | | | | **T1 #18** | | | | | | | | |  |  |  |  |  |
|  | **CLN** | **5** | **3** | **4** | **3** | **4** | **4** | **4** | **6** | **4** | **4** | **5** | **3** | **4** | **3** | **4** | **3** | **7** | **6** | **4** | **3** | **6** | **6** | **5** | **3** | **5** | **4** | **3** | **6** | **3** | **5** | **4** | **5** | **4** | **3** | **4** |  |  | **4.3** | **1.12** | **35** |
|  | **RLN** | **19** | **14** | **14** | **14** | **13** | **12** | **12** | **16** | **14** | **15** | **15** | **14** | **13** | **14** | **13** | **13** | **18** | **17** | **14** | **13** | **17** | **17** | **14** | **15** | **13** | **12** | **13** | **19** | **13** | **16** | **13** | **16** | **14** | **12** | **15** |  |  | **14.5** | **1.93** |  |
| ***35S:GmFT4*** |  | **T1 #1** | | | | | | | | | **T1 #14** | | | | | | | | | **T1 #15** | | | | | |  |  |  |  |  |  |  |  |  |  |  |  |  |  |  |  |
|  | **CLN** | **5** | **7** | **7** | **3** | **7** | **8** | **7** | **10** | **7** | **5** | **10** | **8** | **4** | **9** | **3** | **8** | **4** | **10** | **8** | **7** | **7** | **7** | **7** | **9** |  |  |  |  |  |  |  |  |  |  |  |  |  | **7.0** | **2.05** | **24** |
|  | **RLN** | **21** | **27** | **31** | **14** | **28** | **26** | **27** | **35** | **25** | **22** | **33** | **30** | **24** | **36** | **14** | **33** | **20** | **36** | **33** | **29** | **28** | **25** | **25** | **31** |  |  |  |  |  |  |  |  |  |  |  |  |  | **27.2** | **6.08** |  |
| ***35S:GmFT6*** |  | **T1 #2** | | | | | | | | | **T1 #3** | | | | | | | | | **T1 #6** | | | | | | | | | **T1 #9** | | | | |  |  |  |  |  |  |  |  |
|  | **CLN** | **7** | **5** | **8** | **5** | **15** | **5** | **4** | **6** | **5** | **17** | **6** | **5** | **4** | **5** | **5** | **4** | **20** | **4** | **16** | **15** | **5** | **18** | **4** | **5** | **7** | **14** | **18** | **5** | **4** | **13** | **10** | **6** |  |  |  |  |  | **8.4** | **5.22** | **32** |
|  | **RLN** | **13** | **14** | **22** | **15** | **28** | **13** | **13** | **14** | **16** | **36** | **22** | **13** | **16** | **15** | **15** | **13** | **31** | **16** | **32** | **26** | **17** | **34** | **13** | **16** | **16** | **23** | **34** | **14** | **12** | **21** | **21** | **16** |  |  |  |  |  | **19.4** | **7.33** |  |

*CNL, Cauline leaf number; RLN, Rosette leaf number; AVE, Average; STD, Standard deviation; n, number of plants

**Supplementary Table S4.** Flowering times of soybean landraces determined by the number of days to flowering.

| No. | Germplasm | Flowering time  (days to flowering) | | |
| --- | --- | --- | --- | --- |
|  |  | mean ± s.d.* | range | *n* |
| 1 | Keunolkong | 36.5 ± 1.95 | 34-41 | 14 |
| 2 | IT141493 | 36.9 ± 1.18 | 35-38 | 16 |
| 3 | IT141739 | 37.0 ± 1.10 | 36-39 | 6 |
| 4 | IT22564 | 38.6 ± 1.72 | 36-41 | 15 |
| 5 | IT105996 | 39.2 ± 3.47 | 35-47 | 15 |
| 6 | IT112813 | 42.1 ± 2.31 | 39-45 | 15 |
| 7 | IT113176 | 42.9 ± 2.96 | 39-50 | 15 |
| 8 | IT142886 | 43.6 ± 3.81 | 40-54 | 16 |
| 9 | IT120731 | 45.1 ± 3.34 | 41-52 | 16 |
| 10 | IT110976 | 45.9 ± 1.55 | 41-48 | 13 |
| 11 | IT104062 | 46.7 ± 2.66 | 43-51 | 15 |
| 12 | IT108929 | 47.2 ± 4.39 | 41-55 | 14 |
| 13 | IT143024 | 48.1 ± 2.18 | 45-53 | 14 |
| 14 | IT153757 | 48.6 ±1.86 | 46-52 | 16 |
| 15 | IT153414 | 50.1 ± 2.68 | 46-54 | 16 |
| 16 | IT189252 | 51.6 ± 2.78 | 37-57 | 16 |
| 17 | IT156262 | 52.5 ± 1.88 | 44-57 | 15 |
| 18 | IT186230 | 53.6 ± 1.79 | 49-56 | 14 |
| 19 | IT142915 | 54.5 ± 2.22 | 48-59 | 16 |
| 20 | IT115802 | 55.4 ± 2.97 | 47-61 | 15 |
| 21 | IT115761 | 58.5 ± 2.83 | 54-64 | 15 |
| 22 | IT156217 | 60.3 ± 2.87 | 52-68 | 16 |
| 23 | IT115692 | 60.5 ± 1.92 | 58-64 | 15 |
| 24 | IT115704 | 62.3 ± 3.31 | 55-70 | 15 |

*Data shown in this column indicate mean ± standard deviation.

**Supplementary Table S5.** Primers used for exon swapping.

| Gene | Primer name^*^ | Primer sequence^**^ |
| --- | --- | --- |
| CG4222  CG2444  CG2422  CG4244  CG2242  CG4424  CG2224  CG4442  CG4422  CG2244  CG2222B4  CG4444B2 | MG-1834 (R)  MG-1835 (F)  MG-1836 (R)  MG-1837 (F)  MG-1740 (R)  MG-1741 (F)  MG-1836 (R)  MG-1837 (F)  MG-1735 (R)  MG-1736 (F)  MG-1834 (R)  MG-1835 (F)  MG-1735 (R)  MG-1736 (F)  MG-1737 (R)  MG-1732 (R)  MG-1733 (F)  MG-1740 (R)  MG-1741 (F)  MG-1732 (R)  MG-1733 (F)  MG-1737 (R)  MG-1738 (F)  MG-1740 (R)  MG-1741 (F)  MG-1735 (R)  MG-1736 (F)  MG-1778 (R)  MG-1779 (F)  MG-1780 (R)  MG-1781 (F)  MG-1782 (R)  MG-1783 (F)  MG-1784 (R)  MG-1785 (F) | CAACCGCAATCAGAGTGTAAAAGATCCCTAGGTCAT  TTACACTCTGATTGCGGTTGATCCCGAT  TCACCATTATCAAAGTATAGAAGTTCCTGAGGTCATC  CTATACTTTGATAATGGTGAACCCCGATGC  AGTCACCAACCAATGCAAATATTCCTTCATATGTGGG  ATTTGCATTGGTTGGTGACTGATATCCCAGCAAC  TCACCATTATCAAAGTATAGAAGTTCCTGAGGTCATC  CTATACTTTGATAATGGTGAACCCCGATGC  GGTTACCAACCAATGGAGGTATTCTCTCAAATTGG  ACCTCCATTGGTTGGTAACCAATATTCCAGCATCTA  CAACCGCAATCAGAGTGTAAAAGATCCCTAGGTCAT  TTACACTCTGATTGCGGTTGATCCCGAT  GGTTACCAACCAATGGAGGTATTCTCTCAAATTGG  ACCTCCATTGGTTGGTAACCAATATTCCAGCATCTA  ACCTCATGGCCAGTGGTTGCCCCTGTAGATGCT  ATCTCTTCTCCGAAACTAGCCCCTGTTGTTGCTG  GCTAGTTTCGGAGAAGAGATTGTGGAGTATGAAAGTCC  AGTCACCAACCAATGCAAATATTCCTTCATATGTGGG  ATTTGCATTGGTTGGTGACTGATATCCCAGCAAC  ATCTCTTCTCCGAAACTAGCCCCTGTTGTTGCTG  GCTAGTTTCGGAGAAGAGATTGTGGAGTATGAAAGTCC  ACCTCATGGCCAGTGGTTGCCCCTGTAGATGCT  GCAACCACTGGCCATGAGGTTGTAACATATGAAAGT  AGTCACCAACCAATGCAAATATTCCTTCATATGTGGG  ATTTGCATTGGTTGGTGACTGATATCCCAGCAAC  GGTTACCAACCAATGGAGGTATTCTCTCAAATTGG  ACCTCCATTGGTTGGTAACCAATATTCCAGCATCTA  TCTATCAAATTGACGAAATAACACAAACACCAAA  TATTTCGTCAATTTGATAGACAAATTGTGCATGCT  TAGTGTTGAAATTTTGGCGCCATCTTGGAGCATG  GCGCCAAAATTTCAACACTAAAGAATTTGCTGAACTT  CCTACCCAGTTGACGAAACAACACAAAAGCAATC  TGTTTCGTCAACTGGGTAGGGAGACCGTGTATGCGTC  TAGTGTTGAAATTCTGGCGCCATCCTGGTGCATA  GCGCCAGAATTTCAACACTAGAGATTTTGCTGAGGTT |

*F and R in parentheses indicate forward and reverse, respectively.

**Primer sequence is written from 5’ to 3’.

**Supplementary Table S6.** Primers used for amino acid substitution.

| Gene | Primer name^*^ | Primer sequence^**^ |
| --- | --- | --- |
| *GmFT4(F124L)*  *GmFT4(D125G)*  *GmFT4(Q127E)*  *GmFT4(I128T)*  *GmFT(H130Y)*  *GmFT(R133G)* | MG-2377 (F)  MG-2378 (R)  MG-2379 (F)  MG-2380 (R)  MG-2381 (F)  MG-2382 (R)  MG-2383 (F)  MG-2384 (R)  MG-2385 (F)  MG-2386 (R)  MG-2387 (F)  MG-2388 (R) | GCTTTTGTGTTGTTTCGTCAACTCGATAGACAAATTGTGCATGC  GCATGCACAATTTGTCTATCGAGTTGACGAAACAACACAAAAGC  GTGTTGTTTCGTCAATTTGGGAGACAAATTGTGCATGCTCCAAG  CTTGGAGCATGCACAATTTGTCTCCCAAATTGACGAAACAACAC  GTTTCGTCAATTTGATAGAGAAATTGTGCATGCTCCAAGATGGCG  CGCCATCTTGGAGCATGCACAATTTCTCTATCAAATTGACGAAAC  CGTCAATTTGATAGACAAACTGTGCATGCTCCAAGATGGCGCC  GGCGCCATCTTGGAGCATGCACAGTTTGTCTATCAAATTGACG  CGTCAATTTGATAGACAAATTGTGTACGCTCCAAGATGGCGCC  GGCGCCATCTTGGAGCGTACACAATTTGTCTATCAAATTGACG  GACAAATTGTGCATGCTCCAGGATGGCGCCAAAATTTCAACAC  GTGTTGAAATTTTGGCGCCATCCTGGAGCATGCACAATTTGTC |

*F and R in parentheses indicate forward and reverse, respectively.

**Primer sequence is written from 5’ to 3’.
